# Supplementary material for: How do diverse low-income and middle-income countries implement primary healthcare team integration to support the delivery of comprehensive primary health care? A mixed-methods study protocol from India, Mexico and Uganda
Source: BMJ Open. 2022 May 24;12(5):e055218. doi: 10.1136/bmjopen-2021-055218 (PMC9134158; doi:10.1136/bmjopen-2021-055218)
Supplement: Supplementary data [file bmjopen-2021-055218supp001.pdf]

In-depth interview guide for Community Health Workers

Main question/exploratory questions	Probing questions
**Role and recruitment**<br>Could you let know about the process of your recruitment?<br><br>**Role of the Community Health Workers**<br>What is your role/job description?<br><br>What is expected from you in performing this role?	 <br><br><br><br>What service do you provide?<br><br>Any challenges: work load, lack of skill?
**Training**<br>Could you tell me about the training received?<br>Did you get evaluated at the end of your training?<br>Do you receive ongoing training/refreshment training to keep your skills up to date?	Do you feel you had adequate training for MCH, infectious and chronic diseases prevention and management?<br><br>How confident do you feel to provide chronic diseases services?
**Accreditation**<br>Is your training/ course accredited?	 Is there any system of accreditation?
**Equipment and Supplies**<br>Do you have supplies and equipment you need for providing services?	What about the equipment for the chronic diseases care?<br>Do you receive protocols and guidelines for implementation of the programmes?<br>Do you use any digital technologies like mobile apps or SMS to assist your work?
**Supervision**<br>Can you describe how you are being supervised?<br><br>Do you feel supported? E.g. if there is any error committed in the field, is there someone you can turn easily?	Who supervises you?<br>How often does your supervisor visit you? Is it enough?<br>What does your supervisor do when he/she visits you?<br>o Observation of service delivery

Joshi R, et al. BMJ Open 2022; 12:e055218. doi: 10.1136/bmjopen-2021-055218

	○ Coaching and skills development<br>○ Trouble shooting, problem solving<br>○ Record Review<br>○ Supply check<br>Do you receive any feedback regarding your supervision?<br>Do you think this type of supervision is helpful?
**Team**<br>Do you feel like you are part of a larger team?	How often do you interact with colleagues in the Primary health centre?
**Incentives**<br>What do you think about your remuneration?<br><br>What are the common reasons that Community Health Workers leave their work?<br>What incentives do you think would motivate Community Health Workers and retain them in their job?	Salaries are consistently paid on time.<br>Increase in salary based on performance?<br><br>Ask about administrative reasons (work-related reasons), financial reasons, social reasons (family issue, pursue higher education, being not recognized or valued by the community)
**Community Involvement** Does the community provide any support to you?<br>Do you engage the community in chronic diseases services provision?	Do they provide feedback, support (like financial/gifts in kind), formal recognition/appreciation?
**Opportunity for advancement**<br>What are the opportunities for further promotion or professional advancement in your career?	In what way do you prefer to pursue your career development?
**Data**<br>Tell me about how the information management system works in your facility	What do you do with the data you collect on chronic diseases in your facility?<br>What process is in place to ensure quality of data and provide assistance when needed?
**Linkages to Health System**<br>**Referral System**<br>How do you refer patients for health services you do not or cannot provide?	Do you need to complete a referral form for the client to take to the facility?

Joshi R, *et al. BMJ Open* 2022; 12:e055218. doi: 10.1136/bmjopen-2021-055218

- What about for chronic diseases?	Do you receive any feedback or counter referral from the facility for patients you have referred?<br><br>How do you refer patient with cardiovascular diseases/ Diabetes/ suspected chronic disease cases?
**General questions**<br>What are your biggest challenges as a Community Health Worker?	What changes are needed to help you do your job better?

Joshi R, *et al. BMJ Open* 2022; 12:e055218. doi: 10.1136/bmjopen-2021-055218
