# Supplementary material for: How do diverse low-income and middle-income countries implement primary healthcare team integration to support the delivery of comprehensive primary health care? A mixed-methods study protocol from India, Mexico and Uganda
Source: BMJ Open. 2022 May 24;12(5):e055218. doi: 10.1136/bmjopen-2021-055218 (PMC9134158; doi:10.1136/bmjopen-2021-055218)
Supplement: Supplementary data [file bmjopen-2021-055218supp002.pdf]

In-depth interview guide with health care workers working in the primary healthcare facilities

Main question/exploratory questions	Probing questions
**Role and recruitment**<br>In your opinion, what kind of strategies are needed to ensure delivery of comprehensive primary health care?	What is your view about the role of Community Health Workers in chronic disease control?
**Training**<br>What is your view about Community Health Workers' training?	Do you think Community Health Workers have sufficient knowledge and skill to implement comprehensive primary health care?
**Equipment and Supplies**<br>Do you think there is enough equipment and supplies to adequately manage acute and chronic diseases at Primary Health Centre level	Are protocols, guidelines for the chronic disease programs adequately implemented?<br><br>What are the challenges to improving adherence to guidelines and protocols?<br><br>What kind of equipment and supplies should be provided?
**Supervision**<br>What is your view of the Community Health Workers supervision process?	What is your/your facilities role in the Community Health Workers' supervision?<br><br>What kind of supervision should be in place for Community Health Workers to strengthen the delivery of comprehensive primary health care?
**Incentives**<br>What factors do you think influence Community Health Workers to remain in or leave their job?	Administrative reasons, financial reasons, social reasons
**Community Involvement** How involved is the community in the management of the Health Facility?	Why/ why not?

**Data**<br>Are the information management systems in your facility adequate?	What is needed to overcome challenges to better information management?<br>Who is responsible for data management?<br>How are the data from your facility managed?
**Linkages to Health System Referral System**<br>Can you describe the referral processes?<br><br>What is the challenge for having a good linkage between the health post and the health services?	Are referral guidelines for Community Health Worker?<br><br>What would you recommend for improving linkage of services from the community to the primary healthcare system?
**Integration of chronic diseases program**<br>How integrated are chronic disease programmes with other activities in your Primary Health Centre?	What is your recommendation for effective integration of chronic disease services in the Primary Health Centre settings?<br>What will be the challenges?
