# Supplementary material for: How do diverse low-income and middle-income countries implement primary healthcare team integration to support the delivery of comprehensive primary health care? A mixed-methods study protocol from India, Mexico and Uganda
Source: BMJ Open. 2022 May 24;12(5):e055218. doi: 10.1136/bmjopen-2021-055218 (PMC9134158; doi:10.1136/bmjopen-2021-055218)
Supplement: Supplementary data [file bmjopen-2021-055218supp003.pdf]

In-depth interview guide with policy makers (National & regional heads, program coordinators)

Main question/exploratory questions	Probing questions
**Role and recruitment**<br>How are the following cadres of Primary Health Care workers recruited?<br>- Doctors<br>- Nurses/midwives<br>- Community Health Workers (Community Health Officers, Community Health Extension Workers and Junior Community Health Extension Workers)<br><br>What is expected from Primary Health Care workers to delivery comprehensive primary health care (including NCDs)<br>How do you support Primary Health Care workers to deliver high quality service?	Who selects them? How?<br>How are they posted?
**Training**<br>What is your view about Community Health Worker's training in the implementation of comprehensive primary health care?<br><br>What are the challenges faced in training the Primary Health Care workers to provide comprehensive primary health care?<br><br>What is the plan for their continuous training to equip the Primary Health Care workers to deliver comprehensive primary health care	Do you think the training is adequate to deliver comprehensive primary health care? (communicable and non-communicable diseases prevention and management?<br>Community Health Workers: How do you evaluate the quality of the training given by the school of health technologies health sciences colleges? (modify this based on the country's CHW program)<br>Do you think the Community Health Workers have a capacity to provide care for conditions other than MCH?<br>What plans are there to teach new skills on a regular basis (e.g. fortnightly or quarterly mentorship or on-the-job training)?

**Accreditation**<br>What is the system of accreditation for the level Community Health Workers?	How is the accreditation system implemented? What happens if minimum standards are not met prior to practicing? How could accreditation be improved?
**Equipment and Supplies**<br>Do you think there is optimal provision of equipment and supplies for the Primary Health Care workers to provide comprehensive primary health care?<br><br>What challenges do you think health facilities faced in delivering services?	What protocols, guidelines, and other teaching aid are available for the non-communicable diseases program at the health facility level?
**Supervision**<br>How are the Primary Health Care workers supervised	What is the role of the Federal/State/LGAs in the supervision of the Primary Health Care workers?<br>Is there any particular strategy implemented for the supervision process?<br>Which activities are focussed on during Is there a system to provide supervision feedback/ reports to the Primary Health Cares?
**Team work**	
How is work distributed among the team?	Are tasks shared between different cadres of the workforce?
**Incentives**<br>How are Primary Health Care workers remunerated for their work?<br><br>**Retention and motivation**<br>What are the common reasons why Primary Health Care workers leave their work?<br>What strategies are in place to reduce the attrition of the Primary Health Care workers and ensure adequate distribution, and improve their motivation?	Are salaries processed on time?<br><br>Are there any challenges with payment processes?<br><br>Are there any additional incentive for implementing non-communicable diseases related or other programme activities<br><br>Retention and motivation<br>- Reasons: administrative reasons (work-related reasons), financial reasons, social reasons (family issue, pursue higher education, not recognized or valued by the community)
**Community Involvement** What strategies are in place for the community to support the Community Health Worker in the implementation of NCD programmes?	- What work has been done/planned to introduce the non-communicable diseases implementation role of

	Community Health Workers to the community?
**Opportunity for advancement**<br>What are the opportunities for further promotion or professional advancement through the Primary Health Care workers?	What are the challenges on to achieving this?<br><br>Are there any method to evaluate individual performance?<br><br>Is performance linked with opportunities for career progression?
**Data**<br>Are any strategies being used to improve information management systems through at the Primary Health Care?	How do supervisors monitor the quality of documents and provide assistance?<br>How are information transmitted from the health facility to other part of the health system
**Linkages to Health System**<br>Referral System:<br>How does the referral system work?	Do you think is there a clear referral guideline for the Primary Health Care workers to refer patients?<br>- Are there feedback mechanisms in place<br>- Any arrangement for the emergency conditions
**General questions**<br>What are your biggest challenges for delivering Comprehensive Primary Health Care?	What changes would you recommend?
