# Supplementary material for: How do diverse low-income and middle-income countries implement primary healthcare team integration to support the delivery of comprehensive primary health care? A mixed-methods study protocol from India, Mexico and Uganda
Source: BMJ Open. 2022 May 24;12(5):e055218. doi: 10.1136/bmjopen-2021-055218 (PMC9134158; doi:10.1136/bmjopen-2021-055218)
Supplement: Author's manuscript [file bmjopen-2021-055218.draft_revisions.pdf]

BMJ Open

BMJ Open is committed to open peer review. As part of this commitment we make the peer review history of every article we publish publicly available.

When an article is published we post the peer reviewers' comments and the authors' responses online. We also post the versions of the paper that were used during peer review. These are the versions that the peer review comments apply to.

The versions of the paper that follow are the versions that were submitted during the peer review process. They are not the versions of record or the final published versions. They should not be cited or distributed as the published version of this manuscript.

BMJ Open is an open access journal and the full, final, typeset and author-corrected version of record of the manuscript is available on our site with no access controls, subscription charges or pay-per-view fees (http://bmjopen.bmj.com).

If you have any questions on BMJ Open's open peer review process please email info.bmjopen@bmj.com

BMJ Open

How do diverse low- and middle-income countries implement primary health care team integration to support the delivery of comprehensive primary health care? A mixed methods study protocol from India, Mexico and Uganda

Journal:	*BMJ Open*
Manuscript ID	bmjopen-2021-055218
Article Type:	Protocol
Date Submitted by the Author:	08-Jul-2021
Complete List of Authors:	Joshi, Rohina; The George Institute for Global Health, Health Systems Science; UNSW,<br>Besigye, Innocent; Makerere University<br>Heredia, Ileana; INSP<br>Sharma, Manushi; George Institute for Global Health<br>Peiris, David; The George Institute for Global Health; UNSW,<br>Mash, Robert; Stellenbosch University, Family Medicine and Primary Care<br>Reyes, Hortensia; Instituto Nacional de Salud Pública, Centro de Investigación en Sistemas de Salud<br>Goodyear-Smith, Felicity; The University of Auckland, General Practice & Primary Health care; University of Auckland<br>John, Renu; George Institute for Global Health<br>Ortega-Altamirano, Doris; National Institute of Public Health<br>Orozco, Emanuel ; Instituto Nacional de Salud Publica<br>Ávila-Burgos, Leticia; Instituto Nacional de Salud Publica<br>Jeyakumar, Ragavi; UNSW; The George Institute for Global Health<br>Serván-Mori, Edson; National Institute of Public Health, Centre for Health Systems Research<br>Upadhyaya, Sanjeev; UNICEF<br>Arora, Varun; Post Graduate Institute of Medical Science<br>Praveen, D; The George Institute for Global Health, Research and Development; UNSW
Keywords:	PRIMARY CARE, PUBLIC HEALTH, HEALTH SERVICES ADMINISTRATION & MANAGEMENT

SCHOLARONE™
Manuscripts

How do diverse low- and middle-income countries implement primary health care team integration to support the delivery of comprehensive primary health care? A mixed methods study protocol from India, Mexico and Uganda

Short Title: PHC Team Integration in LMICs

Author list:

Rohina Joshi, The George Institute for Global Health, UNSW, Sydney, Australia; The George Institute for Global Health, New Delhi, India.

Innocent Besigye, Makerere University, Uganda

Ileana B. Heredia-Pi MD, MSc, PhD, Center for Health System Research, National Institute of Public Health, Cuernavaca, Morelos, Mexico. ORCID iD: 0000-0002-9998-9239

Manushi Sharma, MBA, The George Institute for Global Health, New Delhi, India. ORCID iD: 0000-0001-9450-4792

David Peiris, The George Institute for Global Health, UNSW, Sydney, Australia

Robert J Mash, Stellenbosch University, South Africa

Hortensia Reyes-Morales DrSc, Center for Health System Research, the National Institute of Public Health in Mexico, Cuernavaca, Morelos, Mexico. ORCID iD: 0000-0002-9763-4143

Felicity Goodyear-Smith, The University of Auckland, New Zealand

Renu John, The George Institute for Global Health, New Delhi, India

Doris V. Ortega-Altamirano PhD, Center for Health System Research, National Institute of Public Health, Cuernavaca, Morelos, Mexico. ORCID iD: 0000-0003-4767-8268

Emanuel Orozco-Núñez MSc, Center for Health System Research, National Institute of Public Health, Cuernavaca, Morelos, Mexico ORCID iD: 0000-0002-6550-7385

Leticia Ávila-Burgos DrSc , Center for Health System Research, National Institute of Public Health, Cuernavaca, Morelos, Mexico. ORCID iD: 0000-0002-8292-7103

Ragavi Jeyakumar, The George Institute for Global Health, UNSW, Sydney, Australia

Edson Serván-Mori PhD, Center for Health System Research, National Institute of Public Health, Cuernavaca, Morelos, Mexico. ORCID iD: 0000-0001-9820-8325

Sanjeev Upadhyaya, UNICEF Office for Andhra Pradesh, Telangana & Karnataka, India

Varun Arora, Post Graduate Institute of Medical Science, Rohtak, India

Devarsetty Praveen, The George Institute for Global Health, New Delhi, India; University of New South Wales, Australia; Prasanna School of Public Health, Manipal Academy of Higher Education, India

Corresponding Author. Rohina Joshi
The George Institute for Global Health, UNSW
PO Box, M201, Missenden Road
Camperdown, NSW, AUS 2050

For peer review only - http://bmjopen.bmj.com/site/about/guidelines.xhtml

For peer review only

For peer review only - http://bmjopen.bmj.com/site/about/guidelines.xhtml

ABSTRACT

Introduction: Attainment of universal health coverage (UHC) is feasible via strengthened primary health systems that are comprehensive, accessible, people-centred, continuous, and coordinated. Having an adequately trained, motivated, and equipped primary healthcare workforce is central to the provision of comprehensive primary health care (CPHC). This study aims to understand the delivery of CPHC via primary health care (PHC) teams in India, Mexico and Uganda.

Methods and analysis: A parallel, convergent mixed-methods study (integration of quantitative and qualitative results) will be conducted to gain an understanding of PHC teams. Methods include: a) Policy review on PHC team composition, organisation and expected comprehensiveness of PHC services, b) PHC facility review using the WHO Service Availability and Readiness Assessment (SARA), and c) PHC key informant interviews. Data will be collected from 20, 10, and 10 PHCs in India, Mexico and Uganda respectively and analysed using descriptive methods and thematic analysis approach. Outcomes will include an in-depth understanding of the health policies for PHC as well as understanding PHC team composition, organisation and the delivery of comprehensive PHC.

Ethics and dissemination: Approvals have been sought from the Institutional Ethics Committee of The George Institute for Global Health, India for the Indian sites, School of Medicine Research Ethics Committee at Makerere University for the sites in Uganda and the Research, Ethics and Biosecurity Committees of the Mexican National Institute of Public Health for the sites in Mexico. Results will be shared through presentations with governments, publications in peer-reviewed journals and presentations at conferences.

Strengths and limitations:

- This study will provide insight into the availability of policies for PHC workforce in three diverse countries.
- It will help understand the implementation of policies on PHC workforce, and team organisation for the delivery of comprehensive primary health care.

For peer review only

- While each country includes sites from regions representative of the health system of the country, the results are not generalisable beyond the region due to the wide variation in socio-demographic factors and health system structure.

For peer review only

For peer review only - http://bmjopen.bmj.com/site/about/guidelines.xhtml

INTRODUCTION

Primary health care (PHC) in many low- and middle-income countries (LMICs) is fragmented, selectively disease-oriented, and under-resourced with suboptimal performance.[1-4] There is global recognition of the need to strengthen PHC because it is essential for all to have access to affordable high-quality healthcare which is considered the path towards achieving universal health coverage (UHC), the main target for sustainable development goal (SDG) 3.[5] Appropriate high quality PHC is considered as the most equitable and efficient way to enhance the health of populations.[5-7]

In 2017, the World Health Organisation (WHO) developed a framework on integrated people-centred health services (IPCHS), which called for a fundamental shift in the funding, organisation and management of health services.[1,8] IPCHS encourages 'people-centred', rather than 'disease focussed' and 'siloed' health systems, thereby supporting the progress of countries towards UHC. High quality PHC is people-centred, accessible, coordinated, comprehensive and continuous. PHC describes an approach to health policy and service delivery that includes both primary care services delivered to individuals, and public health services delivered to populations.[9] The delivery of high quality PHC is dependent on the availability of adequately skilled and motivated PHC workforce, and the way in which they function as collaborative teams. Workforce availability in turn depends on the country's PHC workforce policies, funding, remuneration, supportive supervision and professionalisation.

This workforce refers to all occupations of health professionals responsible for organising and delivering PHC,[10] essential to deliver high-quality PHC services.[11-13] In a context of increasing demand for health care, driven by demographic, epidemiological and technological changes, the PHC workforce needs to adapt to these changes.[14]

Policies on the PHC workforce, formation of integrated PHC teams, and the capacity of these teams to deliver high quality PHC varies between countries. It is therefore important to understand how PHC teams are organised, and whether the services delivered are truly comprehensive. We define PHC teams as a structured group of multidisciplinary health workers, co-located in a facility and serving a defined population in the community.[15] We use Barbara Starfield's definition of comprehensiveness which

For peer review only - http://bmjopen.bmj.com/site/about/guidelines.xhtml

refers to the provision of holistic and appropriate care across a broad spectrum of health conditions, across the life span, and treatment modalities.[16] While there is a body of research on PHC systems, recent reviews have indicated knowledge gaps on effective PHC team organisation and service delivery.[3,12,14,17] In particular, given variability in health system contexts, there is a need to investigate how different LMICs organise and integrate their PHC teams to deliver comprehensive care.[3,5]

Against this backdrop, the PHC Research Consortium (PHCRC)[17] commissioned researchers from India, Mexico, and Uganda to study PHC team organisation and delivery of comprehensive PHC services. (See Table 1) The aim of this research is to investigate the relationship between different ways of organising PHC workforce and their delivery of comprehensive PHC in three LMICs: India, Mexico, and Uganda, being three large and diverse countries on different continents. Specific objectives are to:

1. Review the national and subnational policies on PHC team composition and organisation and expected comprehensiveness of PHC service delivery.
2. Describe the actual composition and organisation of PHC teams in the sampled health services.
3. Assess the comprehensiveness of care provided by these teams using the above definition
4. Conduct a comparative analysis of the relationship between PHC team composition and organisation with the delivery of comprehensive PHC across the three countries.

Primary Health Care context in India, Mexico, and Uganda

The definition of PHC used in our study is consistent with the Alma-Ata declaration which includes preventive, promotive, curative, and palliative services available at the lower levels of the health system. This study will be conducted in the context of recent health system reforms, commitment of Ministries of Health to SDG Target 3c (Substantially increase health financing and the recruitment, development, training and retention of the health workforce in developing countries) and attainment of UHC (SDG3.8) through PHC.

For peer review only - http://bmjopen.bmj.com/site/about/guidelines.xhtml

PHC System in **India**: Comprehensive Primary Health Care (CPHC) has always been the essence of the Indian health system policy. The health reforms of 2005 and 2017, and 2018 focussed on the actionable and achievable tasks through which CPHC is being realised. In 2005, the National Health Mission, aimed to strengthen the rural health services and provide financial protection to families below the poverty line. Building on this momentum, in 2017, further reforms were made to put CPHC at the forefront. CPHC was intended to address both communicable and non-communicable diseases through PHC centres with multi-disciplinary teams, and to establish new PHC facilities at the village level. These would then link to the PHC, secondary and tertiary health centres. Finally, in 2018, the Government of India introduced Ayushman Bharat (Universal Health Coverage) comprising two major health initiatives – Health and Wellness centres (Upgradation of existing PHCs and sub centers to provide CPHC) and Pradhan Mantri Jan Arogya Yojana (provision of health cover of Rs. 5 lakhs (~$7000) per family per year for secondary and tertiary care hospitalization) covering the entire spectrum of prevention and promotion along with primary, secondary, and tertiary care.[18]

PHC System in **Mexico**: The Mexican public healthcare sector is organised around a segmented model and is marked by the separation of health-care rights between the insured in the salaried, formal sector of the economy and the offer of health services for the poor and uninsured, the latter organized by the recently created Health Institute for Welfare (Instituto de Salud para el Bienestar or INSABI by its Spanish acronym). All population segments receive their health services through vertically integrated institutions, each of which is responsible for stewardship, financing, and service delivery only for that particular group.[6-8] For example, the Mexican Social Security Institute (IMSS by its Spanish acronym) covers the employees of the formal private sector and employees of the army are covered by the Social Security Institute for the Mexican Armed Forces. Launched in 2015 and still in the early stages of implementation, the government's Comprehensive Health Care Model (MAIS by its Spanish acronym) aims to define and monitor patients' care pathways through the system to ensure timely delivery of quality services.[19] The current federal administration aims to strengthen the national health system through a six-year Sectorial Health Program (2019 – 2024). The pillars of the transformation are universal access to health services and free medicines for the entire population, a new Comprehensive

For peer review only - http://bmjopen.bmj.com/site/about/guidelines.xhtml

Primary Health Care (CPHC-I) model, the reorganisation of the health system moving from decentralised to a centralised system, the strengthening of the national pharmaceutical industry, and promotion of research.

PHC system in **Uganda:** Uganda started implementing health sector reforms in the late 1980s and early 1990s as part of a broader decentralisation policy to restore the health system after the political crises of 1970s. Decentralisation allowed the district authorities to cater to the local needs of the communities in terms of service delivery and strategic planning.[20] This decentralised system is based on the district as an administrative unit, with the local government providing stewardship. PHC follows this decentralised system with multi-layered health care delivery from health centre levels 1 to 4, and the general hospital at the apex. PHC administration is based on a Health Sub-District (HSD) system. Each HSD oversees several lower-level health facilities and provides supportive supervision. PHC is provided by nurses, clinical officers and non-specialist doctors, referred to as medical officers. This PHC approach links with the community through the Village Health Teams, which includes non-trained community members. Each health facility also has community members as members of the health unit management committee as a way of involving the community in the management and delivery of the health services. The aim of the HSD is to improve quality of routine health service delivery, increate equity of access to essential health services and foster community involvement in planning, management and delivery of health care.[21]

For peer review only

METHODS

Conceptual framework

The study will use the Primary Health Care Performance Initiative (PHCPI) conceptual framework (Figure 1), and the research will be based on the service delivery and output domains (availability of effective PHC services and high quality PHC, effective service coverage) with a specific focus on the relationship between comprehensiveness of PHC (one of the key quality related PHC issues) and the composition (availability of groups of PHC providers with diverse education and capabilities) and

For peer review only - http://bmjopen.bmj.com/site/about/guidelines.xhtml

organisation (team-based organisation of care to leverage the distinct expertise of different groups for provision of comprehensive PHC) of PHC teams, and to compare models between countries.[22,23]

Study design

This will be a parallel convergent mixed-methods study, which will combine qualitative and quantitative data in each country and support cross-country comparisons. Empirical data will be collected from PHC settings in a prespecified region from the three countries. It will comprise of three steps as shown in Figure 2.

Site selection

Region and site selection will occur purposively to capture the diversity and needs of the population. Each country will first select the regions, and then sites to represent the health needs and overall health system performance of the regions. Overall, 10 health units will be selected for each country except for India where 20 health units will be selected representing the regions, giving a total of 40 PHC units.

In India, a total of 20 PHCs from two regions (Vizianagaram from Andhra Pradesh, South India and Jhajjar from Haryana, North India) have been chosen. In Mexico, 10 PHCs will be included (three PHCs from Northern region, two from Western region; three from Central region and finally two PHCs from South Region). In Uganda, 10 PHCs will be selected from the Eastern (Tororo district) and Western (Buliisa district) regions and will include one General Hospital. (Figure 3)

Data collection and analysis

Objective 1: To review national and sub-national policies on PHC team composition and organisation and expected comprehensiveness of PHC.

Data collection: A desktop review of published and grey literature documents as well as relevant policy documents will be conducted to identify the government regulations or policies related to PHC workforce.

For peer review only - http://bmjopen.bmj.com/site/about/guidelines.xhtml

Analysis: We will review the policies relating to PHC workforce and extract data to a standardised data collection tool template that uses the PHCPI conceptual framework (figure 1) with the below mentioned categories. 1) Governance and leadership; 2) Government spending on PHC; 3) PHC structure and organisation; 4) PHC workforce; 5) PHC service delivery and 6) PHC performance. Data will then be analysed qualitatively using NVivo software to create a narrative synthesis of the country's policy on the areas of interest.

Objective 2: Describe the actual composition and organisation of PHC teams.

Data collection: A cross-sectional descriptive survey will be conducted in the selected PHC facilities. Data collectors will be trained in WHO's Service Availability and Readiness Assessment (SARA) tool and will complete the questionnaire using electronic devices. SARA is a health facility assessment tool designed to assess the available infrastructure, equipment, and workforce, thereby determining the service availability and readiness of the facility to provide CPHC. Data collection will occur at the PHC unit including its community-based outreach centres (e.g. Health and Wellness Centres in India, at the selected PHC units in Mexico and Health Centres 2 and 3 in Uganda) to understand the PHC infrastructure, composition of PHC teams and the services delivered to the community. Data will be collected on electronic devices using the Open Data Kit (ODK) platform, stored locally on the device, and when internet connectivity is available, uploaded to a central repository/server in respective countries for data analysis. When internet is not available, data from the devices can be manually saved in the central repository.

Analysis: Service availability will be described by three domains: health infrastructure, health workforce and service utilization. Continuous variables will be summarised using either mean (SD) or median (IQR). All categorical variables will be summarised using frequencies and percentages.

Objective 3: Assess the comprehensiveness of care provided by PHC teams.

Data collection: This comprises semi-structured in-depth interviews (IDIs) to explore topics on the role and recruitment of the workforce, and how jobs are shared in the team, training, accreditation, supervision, performance evaluation, incentives, career progression, community involvement, team

For peer review only - http://bmjopen.bmj.com/site/about/guidelines.xhtml

For peer review only

composition, organisation and comprehensiveness of services provided. A purposive sample of participants including PHC workforce (community health workers, nurses, social workers, pharmacy staff, health promoters, primary care doctors), and National/Regional/District level policy makers and PHC managers will be invited for the IDIs. Trained researchers from each country will interview participants in local languages (Telugu, Hindi and English in India; Spanish in Mexico; and English, Ateso, Jopadhola and Runyoro in Uganda) using interview guides described in Appendix 1, 2 and 3. Debriefing sessions with the entire research team will be held each week. Interviews will take place over phone/zoom/skype or in-person depending on the local situation of COVID-19 pandemic and will be audio recorded. Participants will be contacted at the health units or their office (policy makers) and will be interviewed in an area within the unit that meets the appropriate privacy conditions. We aim to conduct up to 60 interviews in each country (180 interviews in total).

Data analysis: Interviews will be transcribed verbatim in-country and transcripts in Hindi, Telugu, Spanish, and Ateso, Jopadhola and Runyoro will be translated to English for analysis. The qualitative data for each country will be coded using NVivo software (QRS International, Vic) and analysed using an inductive approach. Two coders from each country will review and analyse the data. Weekly calls will be set up to discuss the emerging themes with the research team. This approach will enable us to explore and identify the important issues in PHC workforce organisation, composition and comprehensiveness, and will also help us to identify shared challenges and differences across countries.

Triangulation of data: The emergent themes from the qualitative interviews in each country will be interpreted in conjunction with the SARA survey and outputs from the policy analysis. Data integration of the three objectives will help us identify the policy and implementation gaps for each country.

Objective 4: Conduct a comparative analysis of the relationship between PHC team composition and organisation with the delivery of comprehensive PHC across the three countries.

Data analysis: We will use a case-oriented research strategy where each 'case' (country) will be considered analytically as a whole.[24,25] Cross country comparisons will be conducted to understand similarities and differences in PHC related policies, especially in terms of the workforce composition, organisation and service delivery with the intention of *learning about* the different approaches to CPHC

For peer review only - http://bmjopen.bmj.com/site/about/guidelines.xhtml

and PHC workforce organisation, the *context* in which PHC systems exist, and *why* they take the forms they do. The comparison will examine the differences and similarities between PHC policies, organisation, and service delivery in the three countries.[25]

ETHICS

Ethical approvals have been sought from Institutional Ethics Committee of The George Institute for Global Health, India for the Indian sites (Ref 16/2020); School of Medicine Research Ethics Committee at Makerere University for the sites in Uganda (Ref 2020-218); and the Mexican National Institute of Public Health (INSP for its Spanish acronym) ethics review board for the sites in Mexico (Ref: 1726). Additional permissions have been sought from the Uganda National Council for Science and Technology and Tororo and Buliisa District Health Offices and the INSP Research committee. The local health authorities will provide approval for collection of data at the facility level. The respondents will be adequately informed regarding all relevant aspects of the study, including its aim and interview procedures, through a participant information sheet. Respondents who accept to participate in the study will provide signed written informed consent. All participants will be given written participant information sheets prior to consenting to participate in this study. Data collection instruments will be piloted and administered by means of electronic questionnaires on mobile devices.

SIGNIFICANCE

This study will provide insight into the availability of policies on PHC, the implementation of policies on PHC workforce, team organisation and service provision for the delivery of CPHC. Furthermore, it will investigate how different LMICs organise their PHC teams to deliver UHC through comprehensive primary care.

For peer review only

Patient and Public Involvement

Patients or public were not involved in the design, conduct or reporting or dissemination of this protocol.

Author's contribution

The study was designed by RJ, DP, and DeP. The first draft was written by RJ, IB, DeP with inputs from RM, FGS and DP. MS, IBH-P, HR-M, ReJ, ES-M, LA-B, EO-N, DO-A, NJ, OJ, RaJ, SU, VA, DeP provided inputs to the protocol. All authors have read and approved the final manuscript.

Funding

This study is funded by the Bill and Melinda Gates Foundation through the Primary Health Care Research Consortium hosted at the George Institute for Global Health in India. RJ is funded by the National Heart Foundation of Australia (Grant number 102059) and the University of New South Wales Scientia Fellowship (No grant number).

Acknowledgement

We would like to acknowledge the Primary Health Care Research Consortium and the team in Uganda (Midiam Ibáñez-Cuevas, Carlos Chivardi-Moreno, Namatovu Jane, and Onyango Jude) for their contribution. This work was supported, in whole, by the Bill & Melinda Gates Foundation [INV-000970]. Under the grant conditions of the Foundation, a Creative Commons Attribution 4.0 Generic License has already been assigned to the Author Accepted Manuscript version that might arise from this submission.

Declaration of Interests

The researchers declare that they have no conflicts of interest.

For peer review only

REFERENCES

1 Bitton A, Ratcliffe HL, Veillard JH, et al. Primary Health Care as a Foundation for Strengthening Health Systems in Low- and Middle-Income Countries. *J Gen Intern Med* 2017;32(5):566-71.doi:10.1007/s11606-016-3898-5 [published Online First: 13 December 2016]

2 Bitton A, Fifield J, Ratcliffe H, et al. Primary healthcare system performance in low-income and middle-income countries: a scoping review of the evidence from 2010 to 2017. *BMJ Glob Health* 2019;4(Suppl 8):e001551.doi:10.1136/bmjgh-2019-001551

3 Goodyear-Smith F, Bazemore A, Coffman M, et al. Primary care financing: a systematic assessment of research priorities in low- and middle-income countries. *BMJ Glob Health* 2019;4(Suppl 8):e001483.doi:10.1136/bmjgh-2019-001483

4 Stenberg K, Hanssen O, Bertram M, et al. Guide posts for investment in primary health care and projected resource needs in 67 low-income and middle-income countries: a modelling study. *Lancet Glob Health* 2019;7(11):e1500-e10.doi:10.1016/S2214-109X(19)30416-4

5 World Health Organization, United Nations Children's Fund. A vision for primary health care in the 21st century: towards universal health coverage and the Sustainable Development Goals. Geneva: World Health Organization, 2018.

6 World Health Organization. Building the economic case for primary health care: a scoping review. Geneva: World Health Organization, 2018.

7 Angell B, Dodd R, Palagyi A, et al. Primary health care financing interventions: a systematic review and stakeholder-driven research agenda for the Asia-Pacific region. *BMJ Glob Health* 2019;4(Suppl 8):e001481.doi:10.1136/bmjgh-2019-001481

8 Veillard J, Cowling K, Bitton A, et al. Better Measurement for Performance Improvement in Low- and Middle-Income Countries: The Primary Health Care Performance Initiative (PHCPI) Experience of Conceptual Framework Development and Indicator Selection. *Milbank Q* 2017;95(4):836-83.doi:10.1111/1468-0009.12301 [published Online First: 2017/12/12]

For peer review only

9 Muldoon LK, Hogg WE, Levitt M. Primary care (PC) and primary health care (PHC). What is the difference? *Can J Public Health* 2006;97(5):409-11.doi:10.1007/bf03405354 [published Online First: 2006/11/24]

10 World Health Organization. Building the primary health care workforce of the 21st century. Geneva: World Health Organization, 2018.

11 World Bank Group, World Health Organization, Ministry of Finance P.R.C., et al. Deepening Health Reform in China: Building High-Quality and Value-Based Service Delivery. Washington, DC: World Bank, 2016.

12 Rodriguez HP, Rogers WH, Marshall RE, et al. Multidisciplinary primary care teams: effects on the quality of clinician-patient interactions and organizational features of care. *Med Care* 2007;45(1):19-27.doi:10.1097/01.mlr.0000241041.53804.29 [published Online First: 2007/02/07]

13 Afsana K, Alam MA, Chen N, et al. Exemplars in Global Health - Community Health Workers in Bangladesh 2021 [Available from: https://www.exemplars.health/topics/community-health-workers/bangladesh, accessed 8 December 2020].

14 Vasan A, Mabey DC, Chaudhri S, et al. Support and performance improvement for primary health care workers in low- and middle-income countries: a scoping review of intervention design and methods. *Health Policy Plan* 2017;32(3):437-52.doi:10.1093/heapol/czw144 [published Online First: 2016/12/21]

15 Wranik WD, Price S, Haydt SM, et al. Implications of interprofessional primary care team characteristics for health services and patient health outcomes: A systematic review with narrative synthesis. *Health Policy* 2019;123(6):550-63. doi: 10.1016/j.healthpol.2019.03.015 [published Online First: 2019/04/09]

16 Starfield B, Shi L, Macinko J. Contribution of primary care to health systems and health. *Milbank Q* 2005;83(3):457-502. doi: 10.1111/j.1468-0009.2005.00409.x [published Online First: 2005/10/06]

17 Primary Health Care Research Consortium. Primary Health Care Research Consortium (PHCRC) 2020 [Available from: https://phcrc.world/, accessed December 9 2020].

For peer review only

18 Ministry of Health & Family Welfare India. Ayushman Bharat: Comprehensive Primary Health Care through Health and Wellness Centers - Operational Guidelines. New Delhi: Government of India, 2018.

19 González Block MÁ, Reyes Morales H, Cahuana Hurtado L, et al. Mexico: Health system review. *Health Syst Transit* 2020;22(222)

20 Ssengooba F, Rahman SA, Hongoro C, et al. Health sector reforms and human resources for health in Uganda and Bangladesh: mechanisms of effect. *Hum Resour Health* 2007;5(1):3. doi: 10.1186/1478-4491-5-3

21 Murindwa G, Tashobya CK, Kyabaggu JH, et al. Meeting the challenges of decentralised health service delivery in Uganda as a component of broader health sector reforms. In: Christine Kirunga Tashobya, Freddie Ssengooba, Valeria Oliveira Cruz, ed. Health Systems Reforms in Uganda: Processes and Outputs. UK: Health Systems Development Programme, London School of Hygiene & Tropical Medicine 2006:97-109.

22 Primary Health Care Performance Initiative. The PHCPI Conceptual Framework 2018 [Available from: https://improvingphc.org/phcpi-conceptual-framework, accessed 1 June 2021].

23 Primary Health Care Performance Initiative. Team-Based Care Organization 2018 [Available from: https://improvingphc.org/improvement-strategies/facility-organization-and-management/team-based-care-organization, accessed 1 June 2021].

24 Imbrogno S. A case oriented research strategy as a comparative method. *J Int Comp Soc Policy* 1994;10(2):137-48.doi:10.1080/17486839408414909

25 Cacace M, Ettelt S, Mays N, et al. Assessing quality in cross-country comparisons of health systems and policies: towards a set of generic quality criteria. *Health Policy* 2013;112(1-2):156-62.doi:10.1016/j.healthpol.2013.03.020 [published Online First: 2013/05/01]

26 World Bank. GDP per capita, PPP (current international $) 2021 [Available from: https://data.worldbank.org/indicator/NY.GDP.PCAP.PP.CD., accessed 1 June 2021]

For peer review only - http://bmjopen.bmj.com/site/about/guidelines.xhtml

27 World Health Organization. Life expectancy and healthy life expectancy Data by country 2020 [Available from: https://apps.who.int/gho/data/view.main.SDG2016LEXv?lang=en., accessed 1 June 2021]

28 World Health Organization. Maternal mortality Estimates by country 2019 [Available from: https://apps.who.int/gho/data/view.main.1390., accessed 1 June 2021]

29 Mor N, Kalita A. Financing Universal Health Care in India. *SSRN Electronic Journal* 2015 doi:10.2139/ssrn.2606918

30 Lahariya C. Health & Wellness Centers to Strengthen Primary Health Care in India: Concept, Progress and Ways Forward. *Indian J Pediatr* 2020;87(11):916-29.doi:10.1007/s12098-020-03359-z [published Online First: 2020/07/09]

31 Shamah-Levy T, Vielma-Orozco E, Heredia-Hernandez O, et al. Encuesta Nacional de Salud y Nutricion 2018-19: Resultados Nacionales. Cuernavaca, Mexico: Instituto Nacional de Salud Publica, 2020.

For peer review only

For peer review only - http://bmjopen.bmj.com/site/about/guidelines.xhtml

Table 1. Primary Health Care context in India, Mexico and Uganda.

	India	Mexico	Uganda
Population, 2020	1.38 billion	128 million	45 million
GDP per capita, PPP (current international $), 2019 [26]	6996.56	20,944.03	2,284.27
Life expectancy at birth (years), 2019 [27]	70.8	76.0	66.7
Maternal mortality ratio (per 100 000 live births), 2017 [28]	145	33	375
Under-five mortality rate (deaths per 1000 live births), 2020 [28]	35.7	16	57.1
Organization	Three tired system:<br>• Sub-center the most peripheral and first contact point between the community and health system<br>• PHC is the first contact point between village community and the Medical Officer<br>• Community Health Center with specialized medical and paramedical staff is the referral unit for PHCs<br>• Tertiary level includes hospital and medical colleges	Three level public health system in Health Districts:<br>• Community Health Centers with a medical doctor student in social service and a nurse<br>• Integrated Community Health Centers with health personnel, nurses, medical doctors, nutritionist, physical activator, social worker<br>• Secondary level includes general hospitals and staff of medical specialties is the referral unit for CHCs<br>• Tertiary level includes hospital of high specialties	Five-level system with health centres I, II, III, IV and the general hospital being the apex of the PHC system. All this functions with the Health Sub District administrative system
Financing	• In 2015-16, 43% of out of pocket expenditure by households was done on primary care.[29]<br>• National Health Policy 2017 commits a major proportion (>2/3rds) of resources to PHC	• In 2018, 50% of total health spending came from Government schemes and compulsory contributory health care financing schemes, of which 24% was spent on primary care units.<br>• 42% of total health spending was out-of-pocket<br>• Population with household expenditures on health greater than 10% of total household expenditure or income (SDG indicator 3.8.2) 1.5%	Uganda's out of pocket on primary care increased through 38.4% through a period of 2004-2018.<br>The government of Uganda expenditure on health has stagnated at around 9.6% of its GDP with regards to the Abuja declaration of 15%

For peer review only

CPHC	The Health and Wellness Center (HWC) component of Ayushman Bharat Programme aims to provide comprehensive primary health care by upgrading and making 150,000 existing sub-centers and primary health centers functional by December 2022.[18] The first HWC was launched on 14 April 2018 and by 31 March 2020, a total 38,595 AB-HWCs were operational across India.[30]	By 2018, 19% of population have no Universal Health Coverage,[31] and to solve this, The National Health Plan 2018-2024 create the Health Institute for Welfare, component of Mexican Health System, and aims to provide comprehensive primary health care by organizing health districts based in geographical areas by 2024.	The Uganda National Minimum Health Care Package (UNMHCP) comprises of interventions that address major causes of morbidity and mortality both communicable and non-Communicable diseases including disease prevention and health promotion. This package of services is funded by government

For peer review only

Figure 1. Primary Healthcare Performance Initiative (PHCPI) conceptual framework

Figure 2. Mixed-methods study design

Figure 3. Sample level distribution and methodological approach

For peer review only

For peer review only

For peer review only - http://bmjopen.bmj.com/site/about/guidelines.xhtml

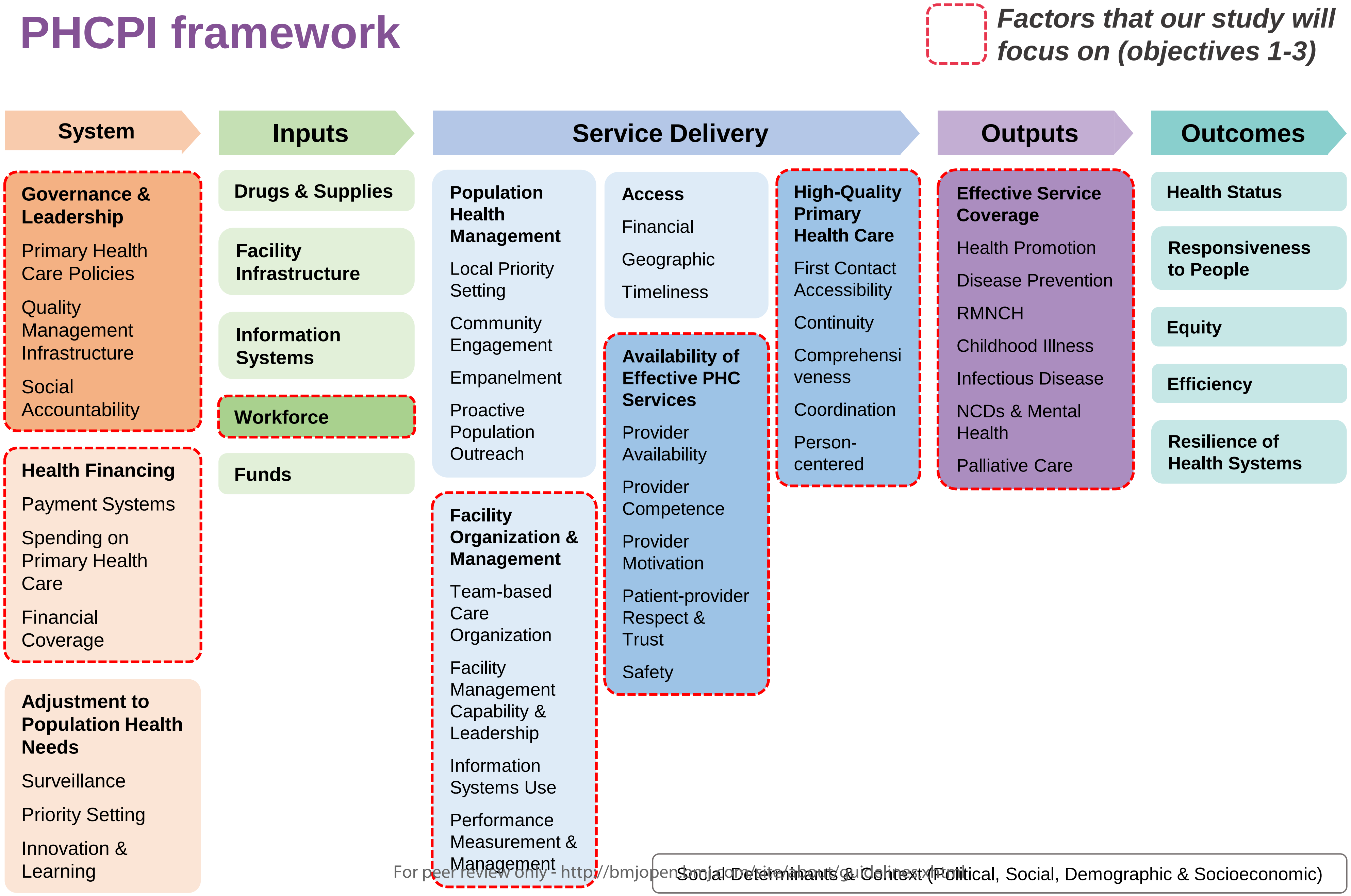
PHCPI framework
Factors that our study will focus on (objectives 1-3)
System
Inputs
Service Delivery
Outputs
Outcomes
Governance & Leadership
Primary Health Care Policies
Quality Management Infrastructure
Social Accountability
Health Financing
Payment Systems
Spending on Primary Health Care
Financial Coverage
Adjustment to Population Health Needs
Surveillance
Priority Setting
Innovation & Learning
Drugs & Supplies
Facility Infrastructure
Information Systems
Workforce
Funds
Population Health Management
Local Priority Setting
Community Engagement
Empanelment
Proactive Population Outreach
Facility Organization & Management
Team-based Care Organization
Facility Management Capability & Leadership
Information Systems Use
Performance Measurement & Management
Access
Financial
Geographic
Timeliness
Availability of Effective PHC Services
Provider Availability
Provider Competence
Provider Motivation
Patient-provider Respect & Trust
Safety
High-Quality Primary Health Care
First Contact Accessibility
Continuity
Comprehensiveness
Coordination
Person-centered
Effective Service Coverage
Health Promotion
Disease Prevention
RMNCH
Childhood Illness
Infectious Disease
NCDs & Mental Health
Palliative Care
Health Status
Responsiveness to People
Equity
Efficiency
Resilience of Health Systems
Social Determinants & Context (Political, Social, Demographic & Socioeconomic)

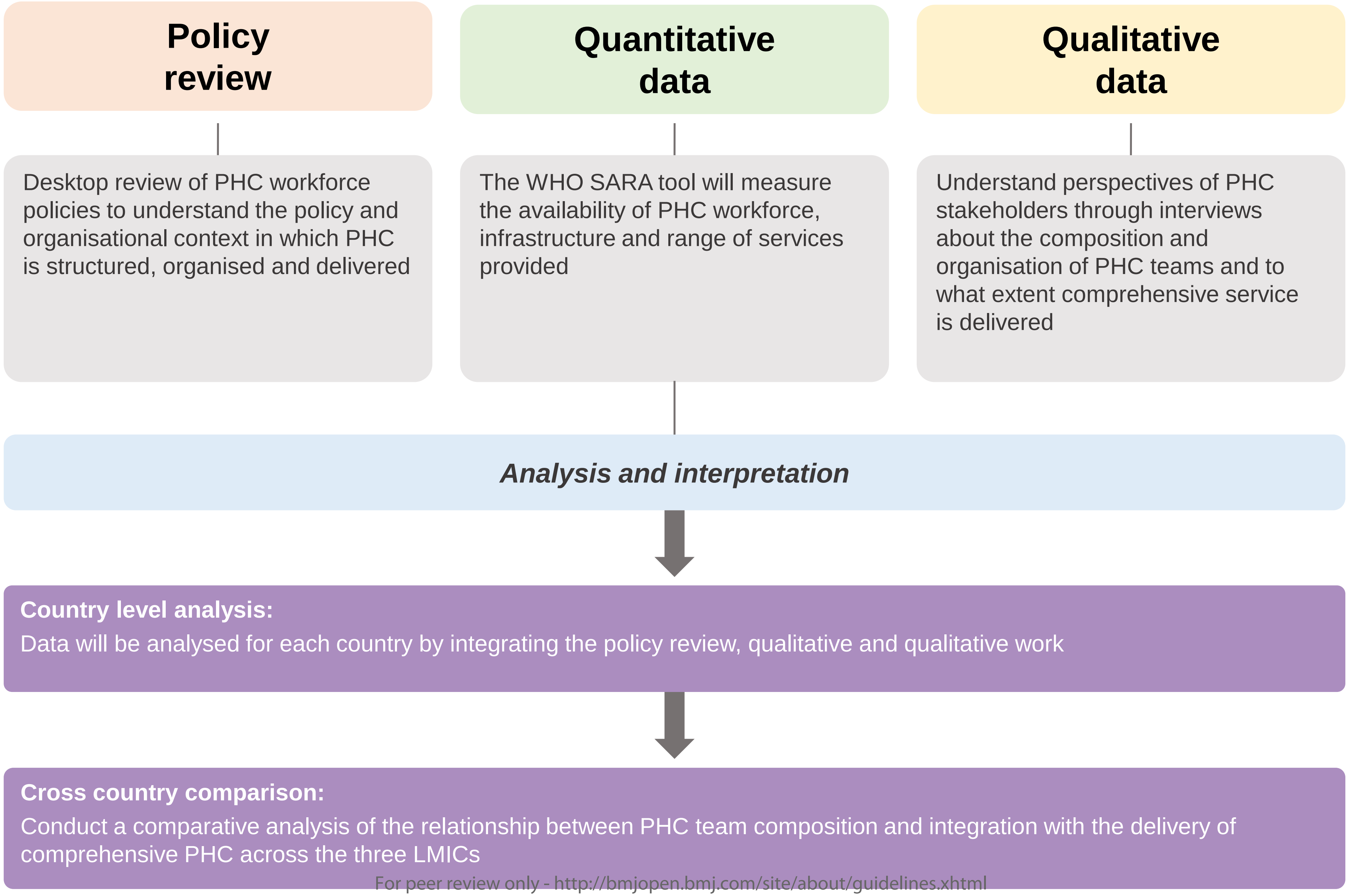
Policy review
Quantitative data
Qualitative data
Desktop review of PHC workforce policies to understand the policy and organisational context in which PHC is structured, organised and delivered
The WHO SARA tool will measure the availability of PHC workforce, infrastructure and range of services provided
Understand perspectives of PHC stakeholders through interviews about the composition and organisation of PHC teams and to what extent comprehensive service is delivered
Analysis and interpretation
Country level analysis:
Data will be analysed for each country by integrating the policy review, qualitative and qualitative work
Cross country comparison:
Conduct a comparative analysis of the relationship between PHC team composition and integration with the delivery of comprehensive PHC across the three LMICs

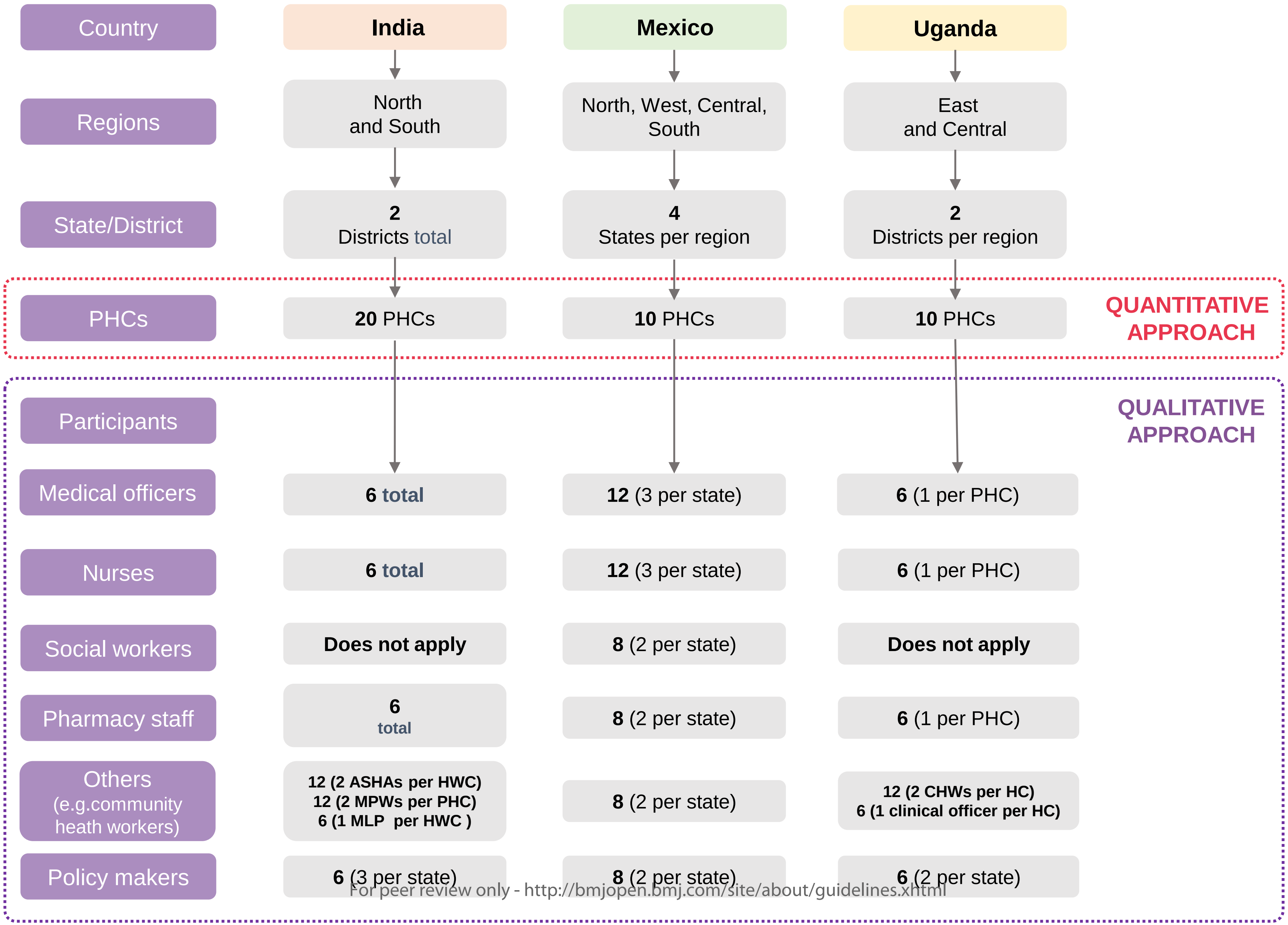

For peer review only - http://bmjopen.bmj.com/site/about/guidelines.xhtml

In-depth interview guide for Community Health Workers

Main question/exploratory questions	Probing questions
**Role and recruitment**<br>Could you let know about the process of your recruitment?<br><br>**Role of the Community Health Workers**<br>What is your role/job description?<br><br>What is expected from you in performing this role?	 <br><br><br>What service do you provide?<br><br>Any challenges: work load, lack of skill?
**Training**<br>Could you tell me about the training received?<br>Did you get evaluated at the end of your training?<br>Do you receive ongoing training/refreshment training to keep your skills up to date?	Do you feel you had adequate training for MCH, infectious and chronic diseases prevention and management?<br><br>How confident do you feel to provide chronic diseases services?
**Accreditation**<br>Is your training/ course accredited?	Is there any system of accreditation?
**Equipment and Supplies**<br>Do you have supplies and equipment you need for providing services?	What about the equipment for the chronic diseases care?<br>Do you receive protocols and guidelines for implementation of the programmes?<br>Do you use any digital technologies like mobile apps or SMS to assist your work?
**Supervision**<br>Can you describe how you are being supervised?<br><br>Do you feel supported? E.g. if there is any error committed in the field, is there someone you can turn easily?	Who supervises you?<br>How often does your supervisor visit you? Is it enough?<br>What does your supervisor do when he/she visits you?<br>○ Observation of service delivery<br>○ Coaching and skills

For peer review only - http://bmjopen.bmj.com/site/about/guidelines.xhtml

	development<br>○ Trouble shooting, problem solving<br>○ Record Review<br>○ Supply check<br>Do you receive any feedback regarding your supervision?<br>Do you think this type of supervision is helpful?
**Team**<br>Do you feel like you are part of a larger team?	How often do you interact with colleagues in the Primary health centre?
**Incentives**<br>What do you think about your remuneration?<br><br>What are the common reasons that Community Health Workers leave their work?<br>What incentives do you think would motivate Community Health Workers and retain them in their job?	Salaries are consistently paid on time.<br>Increase in salary based on performance?<br><br>Ask about administrative reasons (work-related reasons), financial reasons, social reasons (family issue, pursue higher education, being not recognized or valued by the community)
**Community Involvement** Does the community provide any support to you?<br>Do you engage the community in chronic diseases services provision?	Do they provide feedback, support (like financial/gifts in kind), formal recognition/appreciation?
**Opportunity for advancement**<br>What are the opportunities for further promotion or professional advancement in your career?	In what way do you prefer to pursue your career development?
**Data**<br>Tell me about how the information management system works in your facility	What do you do with the data you collect on chronic diseases in your facility?<br>What process is in place to ensure quality of data and provide assistance when needed?
**Linkages to Health System**<br>**Referral System**<br>How do you refer patients for health services you do not or cannot provide?<br>- What about for chronic diseases?	Do you need to complete a referral form for the client to take to the facility?<br>Do you receive any feedback or counter referral from the facility for

For peer review only - http://bmjopen.bmj.com/site/about/guidelines.xhtml

	patients you have referred?<br><br>How do you refer patient with cardiovascular diseases/ Diabetes/ suspected chronic disease cases?
**General questions**<br>What are your biggest challenges as a Community Health Worker?	What changes are needed to help you do your job better?

For peer review only

For peer review only - http://bmjopen.bmj.com/site/about/guidelines.xhtml

In-depth interview guide with health care workers working in the primary healthcare facilities

Main question/exploratory questions	Probing questions
**Role and recruitment**<br>In your opinion, what kind of strategies are needed to ensure delivery of comprehensive primary health care?	What is your view about the role of Community Health Workers in chronic disease control?
**Training**<br>What is your view about Community Health Workers' training?	Do you think Community Health Workers have sufficient knowledge and skill to implement comprehensive primary health care?
**Equipment and Supplies**<br>Do you think there is enough equipment and supplies to adequately manage acute and chronic diseases at Primary Health Centre level	Are protocols, guidelines for the chronic disease programs adequately implemented?<br><br>What are the challenges to improving adherence to guidelines and protocols?<br><br>What kind of equipment and supplies should be provided?
**Supervision**<br>What is your view of the Community Health Workers supervision process?	What is your/your facilities role in the Community Health Workers' supervision?<br><br>What kind of supervision should be in place for Community Health Workers to strengthen the delivery of comprehensive primary health care?
**Incentives**<br>What factors do you think influence Community Health Workers to remain in or leave their job?	Administrative reasons, financial reasons, social reasons
**Community Involvement** How involved is the community in the management of the Health Facility?	Why/ why not?

For peer review only

**Data**<br>Are the information management systems in your facility adequate?	What is needed to overcome challenges to better information management?<br>Who is responsible for data management?<br>How are the data from your facility managed?
**Linkages to Health System Referral System**<br>Can you describe the referral processes?<br><br>What is the challenge for having a good linkage between the health post and the health services?	Are referral guidelines for Community Health Worker?<br><br>What would you recommend for improving linkage of services from the community to the primary healthcare system?
**Integration of chronic diseases program**<br>How integrated are chronic disease programmes with other activities in your Primary Health Centre?	What is your recommendation for effective integration of chronic disease services in the Primary Health Centre settings?<br>What will be the challenges?

For peer review only

In-depth interview guide with policy makers (National & regional heads, program coordinators)

Main question/exploratory questions	Probing questions
**Role and recruitment**<br>How are the following cadres of Primary Health Care workers recruited?<br>- Doctors<br>- Nurses/midwives<br>- Community Health Workers (Community Health Officers, Community Health Extension Workers and Junior Community Health Extension Workers)<br><br>What is expected from Primary Health Care workers to delivery comprehensive primary health care (including NCDs)<br>How do you support Primary Health Care workers to deliver high quality service?	Who selects them? How?<br>How are they posted?
**Training**<br>What is your view about Community Health Worker's training in the implementation of comprehensive primary health care?<br><br>What are the challenges faced in training the Primary Health Care workers to provide comprehensive primary health care?<br><br>What is the plan for their continuous training to equip the Primary Health Care workers to deliver comprehensive primary health care	Do you think the training is adequate to deliver comprehensive primary health care? (communicable and non-communicable diseases prevention and management?<br>Community Health Workers: How do you evaluate the quality of the training given by the school of health technologies health sciences colleges? (modify this based on the country's CHW program)<br>Do you think the Community Health Workers have a capacity to provide care for conditions other than MCH?<br>What plans are there to teach new skills on a regular basis (e.g. fortnightly or quarterly mentorship or on-the-job training)?

For peer review only

**Accreditation**<br>What is the system of accreditation for the level Community Health Workers?	How is the accreditation system implemented? What happens if minimum standards are not met prior to practicing? How could accreditation be improved?
**Equipment and Supplies**<br>Do you think there is optimal provision of equipment and supplies for the Primary Health Care workers to provide comprehensive primary health care?<br><br>What challenges do you think health facilities faced in delivering services?	What protocols, guidelines, and other teaching aid are available for the non-communicable diseases program at the health facility level?
**Supervision**<br>How are the Primary Health Care workers supervised	What is the role of the Federal/State/LGAs in the supervision of the Primary Health Care workers?<br>Is there any particular strategy implemented for the supervision process?<br>Which activities are focussed on during<br>Is there a system to provide supervision feedback/ reports to the Primary Health Cares?
**Team work**	
How is work distributed among the team?	Are tasks shared between different cadres of the workforce?
**Incentives**<br>How are Primary Health Care workers remunerated for their work?<br><br><br><br>**Retention and motivation**<br>What are the common reasons why Primary Health Care workers leave their work?<br>What strategies are in place to reduce the attrition of the Primary Health Care workers and ensure adequate distribution, and improve their motivation?	Are salaries processed on time?<br><br>Are there any challenges with payment processes?<br><br>Are there any additional incentive for implementing non-communicable diseases related or other programme activities<br><br>Retention and motivation<br>- Reasons: administrative reasons (work-related reasons), financial reasons, social reasons (family issue, pursue higher education, not recognized or valued by the community)
**Community Involvement** What strategies are in place for the community to support the Community Health Worker in the implementation of NCD programmes?	- What work has been done/planned to introduce the non-communicable diseases implementation role of

	Community Health Workers to the community?
**Opportunity for advancement**<br>What are the opportunities for further promotion or professional advancement through the Primary Health Care workers?	What are the challenges on to achieving this?<br><br>Are there any method to evaluate individual performance?<br><br>Is performance linked with opportunities for career progression?
**Data**<br>Are any strategies being used to improve information management systems through at the Primary Health Care?	How do supervisors monitor the quality of documents and provide assistance?<br>How are information transmitted from the health facility to other part of the health system
**Linkages to Health System**<br>Referral System:<br>How does the referral system work?	Do you think is there a clear referral guideline for the Primary Health Care workers to refer patients?<br>- Are there feedback mechanisms in place<br>- Any arrangement for the emergency conditions
**General questions**<br>What are your biggest challenges for delivering Comprehensive Primary Health Care?	What changes would you recommend?

For peer review only

BMJ Open

How do diverse low- and middle-income countries implement primary health care team integration to support the delivery of comprehensive primary health care? A mixed methods study protocol from India, Mexico and Uganda

Journal:	*BMJ Open*
Manuscript ID	bmjopen-2021-055218.R1
Article Type:	Protocol
Date Submitted by the Author:	27-Feb-2022
Complete List of Authors:	Joshi, Rohina; University of New South Wales School of Public Health and Community Medicine, Global Health; The George Institute for Global Health India,<br>Besigye, Innocent; Makerere University<br>Heredia, Ileana; INSP<br>Sharma, Manushi; George Institute for Global Health<br>Peiris, David; The George Institute for Global Health; UNSW,<br>Mash, Robert; Stellenbosch University, Family Medicine and Primary Care<br>Reyes, Hortensia; Instituto Nacional de Salud Pública, Centro de Investigación en Sistemas de Salud<br>Goodyear-Smith, Felicity; The University of Auckland, General Practice & Primary Health care; University of Auckland<br>John, Renu; George Institute for Global Health<br>Ortega-Altamirano, Doris; National Institute of Public Health<br>Orozco, Emanuel ; Instituto Nacional de Salud Publica<br>Ávila-Burgos, Leticia; Instituto Nacional de Salud Publica<br>Jeyakumar, Ragavi; UNSW; The George Institute for Global Health<br>Serván-Mori, Edson; National Institute of Public Health, Centre for Health Systems Research<br>Upadhyaya, Sanjeev; UNICEF<br>Arora, Varun; Post Graduate Institute of Medical Science<br>Praveen, D; The George Institute for Global Health, Research and Development; UNSW
**Primary Subject Heading**:	Global health
Secondary Subject Heading:	Health services research, Public health
Keywords:	PUBLIC HEALTH, HEALTH SERVICES ADMINISTRATION & MANAGEMENT, PRIMARY CARE

SCHOLARONE™
Manuscripts

How do diverse low- and middle-income countries implement primary health care team integration to support the delivery of comprehensive primary health care? A mixed methods study protocol from India, Mexico and Uganda

Short Title: PHC Team Integration in LMICs

Author list:

Rohina Joshi, School of Population Health, UNSW, Sydney, Australia; The George Institute for Global Health, New Delhi, India. ORCHID iD: 0000-0002-3374-401X

Innocent K Besigye, Department of Family Medicine, Makerere University, Kampala, Uganda. ORCID iD: 0000-0002-7903-3985

Ileana B. Heredia-Pi MD, MSc, PhD, Center for Health System Research, National Institute of Public Health, Cuernavaca, Morelos, Mexico. ORCID iD: 0000-0002-9998-9239

Manushi Sharma, MBA, The George Institute for Global Health, New Delhi, India. ORCID iD: 0000-0001-9450-4792

David Peiris, The George Institute for Global Health, UNSW, Sydney, Australia ORCID iD 0000-0002-6898-3870

Robert J Mash, Stellenbosch University, South Africa

Hortensia Reyes-Morales DrSc, Center for Health System Research, the National Institute of Public Health in Mexico, Cuernavaca, Morelos, Mexico. ORCID iD: 0000-0002-9763-4143

Felicity Goodyear-Smith, The University of Auckland, New Zealand

Renu John, The George Institute for Global Health, New Delhi, India

Doris V. Ortega-Altamirano PhD, Center for Health System Research, National Institute of Public Health, Cuernavaca, Morelos, Mexico. ORCID iD: 0000-0003-4767-8268

Emanuel Orozco-Núñez MSc, Center for Health System Research, National Institute of Public Health, Cuernavaca, Morelos, Mexico ORCID iD: 0000-0002-6550-7385

Leticia Ávila-Burgos DrSc , Center for Health System Research, National Institute of Public Health, Cuernavaca, Morelos, Mexico. ORCID iD: 0000-0002-8292-7103

Ragavi Jeyakumar, The George Institute for Global Health, UNSW, Sydney, Australia

Edson Serván-Mori PhD, Center for Health System Research, National Institute of Public Health, Cuernavaca, Morelos, Mexico. ORCID iD: 0000-0001-9820-8325

Sanjeev Upadhyaya, UNICEF Office for Andhra Pradesh, Telangana & Karnataka, India

Varun Arora, Post Graduate Institute of Medical Science, Rohtak, India

Devarsetty Praveen, The George Institute for Global Health, New Delhi, India; University of New South Wales, Australia; Prasanna School of Public Health, Manipal Academy of Higher Education, India ORCID iD 0000-0002-0973-943X

Corresponding Author. Rohina Joshi
The George Institute for Global Health, UNSW
PO Box, M201, Missenden Road
Camperdown, NSW, AUS 2050
rjoshi@georgeinstitute.org

For peer review only - http://bmjopen.bmj.com/site/about/guidelines.xhtml

1

For peer review only

For peer review only - http://bmjopen.bmj.com/site/about/guidelines.xhtml

ABSTRACT

Introduction: Attainment of universal health coverage (UHC) is feasible via strengthened primary health systems that are comprehensive, accessible, people-centred, continuous, and coordinated. Having an adequately trained, motivated, and equipped primary healthcare workforce is central to the provision of comprehensive primary health care (CPHC). This study aims to understand PHC team integration, composition and organization in the delivery of CPHC in India, Mexico and Uganda.

Methods and analysis: A parallel, mixed-methods study (integration of quantitative and qualitative results) will be conducted to gain an understanding of PHC teams. Methods include: a) Policy review on PHC team composition, organisation and expected comprehensiveness of PHC services, b) PHC facility review using the WHO Service Availability and Readiness Assessment (SARA), and c) PHC key informant interviews. Data will be collected from 20, 10, and 10 PHCs in India, Mexico and Uganda respectively and analysed using descriptive methods and thematic analysis approach. Outcomes will include an in-depth understanding of the health policies for PHC as well as understanding PHC team composition, organisation and the delivery of comprehensive PHC.

Ethics and dissemination: Approvals have been sought from the Institutional Ethics Committee of The George Institute for Global Health, India for the Indian sites, School of Medicine Research Ethics Committee at Makerere University for the sites in Uganda and the Research, Ethics and Biosecurity Committees of the Mexican National Institute of Public Health for the sites in Mexico. Results will be shared through presentations with governments, publications in peer-reviewed journals and presentations at conferences.

Strengths and limitations:

- This study will provide insight into the availability of policies for PHC workforce in three diverse countries.
- It will help understand the implementation of policies on PHC workforce, and team organisation for the delivery of comprehensive primary health care.

For peer review only - http://bmjopen.bmj.com/site/about/guidelines.xhtml

- While each country includes sites from regions representative of the health system of the country, the results are not generalisable beyond the region due to the wide variation in socio-demographic factors and health system structure.

For peer review only

For peer review only - http://bmjopen.bmj.com/site/about/guidelines.xhtml

INTRODUCTION

Primary health care (PHC) in many low- and middle-income countries (LMICs) is fragmented, selectively disease-oriented, and under-resourced with suboptimal performance.[1-4] There is global recognition of the need to strengthen PHC because it is essential for all to have access to affordable high-quality healthcare which is considered the path towards achieving universal health coverage (UHC), the main target for sustainable development goal (SDG) 3.[5] Appropriate high quality PHC is considered as the most equitable and efficient way to enhance the health of populations.[5-7]

In 2017, the World Health Organisation (WHO) developed a framework on integrated people-centred health services (IPCHS), which called for a fundamental shift in the funding, organisation and management of health services. [1,8] IPCHS encourages 'people-centred', rather than 'disease focussed' and 'siloed' health systems, thereby supporting the progress of countries towards UHC. High quality PHC is people-centred, accessible, coordinated, comprehensive and continuous. PHC describes an approach to health policy and service delivery that includes both primary care services delivered to individuals, and public health services delivered to populations.[9] The delivery of high quality PHC is dependent on the availability of adequately skilled and motivated PHC workforce, and the way in which they function as collaborative teams. Workforce availability in turn depends on the country's PHC workforce policies, funding, remuneration, supportive supervision and professionalisation.

This workforce refers to all occupations of health professionals responsible for organising and delivering PHC,[10] essential to deliver high-quality PHC services. [11-13] In a context of increasing demand for health care, driven by demographic, epidemiological and technological changes, the PHC workforce needs to adapt to these changes.[14]

Policies on the PHC workforce, formation of integrated PHC teams, and the capacity of these teams to deliver high quality PHC varies between countries. It is therefore important to understand how PHC teams are organised, and whether the services delivered are truly comprehensive. We define PHC teams as a structured group of multidisciplinary health workers, co-located in a facility and serving a defined population in the community.[15] We use Barbara Starfield's definition of comprehensiveness which

For peer review only - http://bmjopen.bmj.com/site/about/guidelines.xhtml

refers to the provision of holistic and appropriate care across a broad spectrum of health conditions, across the life span, and treatment modalities.[16] While there is a body of research on PHC systems, recent reviews have indicated knowledge gaps on effective PHC team organisation and service delivery.[3,12,14,17] In particular, given variability in health system contexts, there is a need to investigate how different LMICs organise and integrate their PHC teams to deliver comprehensive care.[3,5]

Against this backdrop, the PHC Research Consortium (PHCRC)[17] commissioned researchers from India, Mexico, and Uganda to study PHC team organisation and delivery of comprehensive PHC services. (See Table 1) The aim of this research is to investigate the relationship between different ways of organising PHC workforce and their delivery of comprehensive PHC in three LMICs: India, Mexico, and Uganda, being three large and diverse countries on different continents. Specific objectives are to:

1. Review the national and subnational policies on PHC team composition and organisation and expected comprehensiveness of PHC service delivery.
2. Describe the actual composition and organisation of PHC teams in the sampled health services.
3. Assess the comprehensiveness of care provided by these teams using the above definition
4. Conduct a comparative analysis of the relationship between PHC team composition and organisation with the delivery of comprehensive PHC across the three countries.

Primary Health Care context in India, Mexico, and Uganda

The definition of PHC used in our study is consistent with the Alma-Ata declaration which includes preventive, promotive, curative, and palliative services available at the lower levels of the health system. This study will be conducted in the context of recent health system reforms, commitment of Ministries of Health to SDG Target 3c (Substantially increase health financing and the recruitment, development, training and retention of the health workforce in developing countries) and attainment of UHC (SDG3.8) through PHC.

For peer review only

PHC System in **India**: Comprehensive Primary Health Care (CPHC) has always been the essence of the Indian health system policy. The health reforms of 2005 and 2017, and 2018 focussed on the actionable and achievable tasks through which CPHC is being realised. In 2005, the National Health Mission, aimed to strengthen the rural health services and provide financial protection to families below the poverty line. Building on this momentum, in 2017, further reforms were made to put CPHC at the forefront. CPHC was intended to address both communicable and non-communicable diseases through PHC centres with multi-disciplinary teams, and to establish new PHC facilities at the village level. These would then link to the PHC, secondary and tertiary health centres. Finally, in 2018, the Government of India introduced Ayushman Bharat (Universal Health Coverage) comprising two major health initiatives – Health and Wellness centres (Upgradation of existing PHCs and sub centers to provide CPHC) and Pradhan Mantri Jan Arogya Yojana (provision of health cover of Rs. 5 lakhs (~$7000) per family per year for secondary and tertiary care hospitalization) covering the entire spectrum of prevention and promotion along with primary, secondary, and tertiary care.[18]

PHC System in **Mexico**: The Mexican public healthcare sector is organised around a segmented model and is marked by the separation of health-care rights between the insured in the salaried, formal sector of the economy and the offer of health services for the poor and uninsured, the latter organized by the recently created Health Institute for Welfare (Instituto de Salud para el Bienestar or INSABI by its Spanish acronym). All population segments receive their health services through vertically integrated institutions, each of which is responsible for stewardship, financing, and service delivery only for that particular group. [6-8] For example, the Mexican Social Security Institute (IMSS by its Spanish acronym) covers the employees of the formal private sector and employees of the army are covered by the Social Security Institute for the Mexican Armed Forces. Launched in 2015 and still in the early stages of implementation, the government's Comprehensive Health Care Model (MAIS by its Spanish acronym) aims to define and monitor patients' care pathways through the system to ensure timely delivery of quality services.[19] The current federal administration aims to strengthen the national health system through a six-year Sectorial Health Program (2019 – 2024). The pillars of the transformation are universal access to health services and free medicines for the entire population, a new Comprehensive

For peer review only - http://bmjopen.bmj.com/site/about/guidelines.xhtml

Primary Health Care (CPHC-I) model, the reorganisation of the health system moving from decentralised to a centralised system, the strengthening of the national pharmaceutical industry, and promotion of research.

PHC system in **Uganda:** Uganda started implementing health sector reforms in the late 1980s and early 1990s as part of a broader decentralisation policy to restore the health system after the political crises of 1970s. Decentralisation allowed the district authorities to cater to the local needs of the communities in terms of service delivery and strategic planning.[20] This decentralised system is based on the district as an administrative unit, with the local government providing stewardship. PHC follows this decentralised system with multi-layered health care delivery from health centre levels 1 to 4, and the general hospital at the apex. PHC administration is based on a Health Sub-District (HSD) system. Each HSD oversees several lower-level health facilities and provides supportive supervision. PHC is provided by nurses, clinical officers and non-specialist doctors, referred to as medical officers. This PHC approach links with the community through the Village Health Teams, which includes non-trained community members. Each health facility also has community members as members of the health unit management committee as a way of involving the community in the management and delivery of the health services. The aim of the HSD is to improve quality of routine health service delivery, increate equity of access to essential health services and foster community involvement in planning, management and delivery of health care.[21]

METHODS

Conceptual framework

The study will use the Primary Health Care Performance Initiative (PHCPI) conceptual framework (Figure 1), and the research will be based on the service delivery and output domains (availability of effective PHC services and high quality PHC, effective service coverage) with a specific focus on the relationship between comprehensiveness of PHC (one of the key quality related PHC issues) and the composition (availability of groups of PHC providers with diverse education and capabilities) and

For peer review only - http://bmjopen.bmj.com/site/about/guidelines.xhtml

organisation (team-based organisation of care to leverage the distinct expertise of different groups for provision of comprehensive PHC) of PHC teams, and to compare models between countries.[22,23]

Study design

This will be a parallel mixed-methods study, which will combine qualitative and quantitative data in each country and support cross-country comparisons. Empirical data will be collected from PHC settings in a prespecified region from the three countries. It will comprise of three steps as shown in Figure 2.

Patient and Public Involvement

Patients or public were not involved in the design, conduct or reporting or dissemination of this protocol.

Site selection

Region and site selection will occur purposively to capture the diversity and needs of the population. Each country will first select the regions, and then sites to represent the health needs and overall health system performance of the regions. Overall, 10 health units will be selected for each country except for India where 20 health units will be selected representing the regions, giving a total of 40 PHC units.

In India, a total of 20 PHCs from two regions (Vizianagaram from Andhra Pradesh, South India and Jhajjar from Haryana, North India) have been chosen. In Mexico, 10 PHCs will be included (three PHCs from Northern region, two from Western region; three from Central region and finally two PHCs from South Region). In Uganda, 10 PHCs will be selected from the Eastern (Tororo district) and Western (Buliisa district) regions and will include one General Hospital. (Figure 3)

Data collection and analysis

Objective 1: To review national and sub-national policies on PHC team composition and organisation and expected comprehensiveness of PHC.

For peer review only

For peer review only - http://bmjopen.bmj.com/site/about/guidelines.xhtml

Data collection: A desktop review of published and grey literature documents as well as relevant policy documents will be conducted to identify the government regulations or policies related to PHC workforce.

Analysis: We will review the policies relating to PHC workforce and extract data to a standardised data collection tool template that uses the PHCPI conceptual framework (figure 1) with the below mentioned categories. 1) Governance and leadership; 2) Government spending on PHC; 3) PHC structure and organisation; 4) PHC workforce; 5) PHC service delivery and 6) PHC performance. Data will then be analysed qualitatively using NVivo software to create a narrative synthesis of the country's policy on the areas of interest.

Objective 2: Describe the actual composition and organisation of PHC teams.

Data collection: A cross-sectional descriptive survey will be conducted in the selected PHC facilities. Data collectors will be trained in WHO's Service Availability and Readiness Assessment (SARA) tool and will complete the questionnaire using electronic devices. SARA is a health facility assessment tool designed to assess the available infrastructure, equipment, and workforce, thereby determining the service availability and readiness of the facility to provide CPHC. We will not collect information about the availability of medicines at the PHC level as this study is focussing on health workforce. Data collection will occur at the PHC unit including its community-based outreach centres (e.g. Health and Wellness Centres in India, at the selected PHC units in Mexico and Health Centres 2 and 3 in Uganda) to understand the PHC infrastructure, composition of PHC teams and the services delivered to the community. Data will be collected on electronic devices using the Open Data Kit (ODK) platform, stored locally on the device, and when internet connectivity is available, uploaded to a central repository/server in respective countries for data analysis. When internet is not available, data from the devices can be manually saved in the central repository.

Analysis: Service availability will be described by three domains: health infrastructure, health workforce and service utilization. Continuous variables will be summarised using either mean (SD) or median (IQR). All categorical variables will be summarised using frequencies and percentages.

For peer review only

For peer review only - http://bmjopen.bmj.com/site/about/guidelines.xhtml

Objective 3: Assess the comprehensiveness of care provided by PHC teams.

Data collection: This comprises semi-structured in-depth interviews (IDIs) to explore topics on the role and recruitment of the workforce, and how jobs are shared in the team, training, accreditation, supervision, performance evaluation, incentives, career progression, community involvement, team composition, organisation and comprehensiveness of services provided. Comprehensiveness of services will be assessed by asking which services are delivered, the range of conditions addressed by the team, if the workforce is trained in managing those conditions, and do the range of services include prevention, promotion, treatment, rehabilitation and palliation? For instance, does that PHC provide care for cardiovascular risk factors and if so, are the staff trained and do they have access to the necessary equipment to measure the risk factors? A purposive sample of participants including PHC workforce (community health workers, nurses, social workers, pharmacy staff, health promoters, primary care doctors), and National/Regional/District level policy makers and PHC managers will be invited for the IDIs. Trained researchers from each country will interview participants in local languages (Telugu, Hindi and English in India; Spanish in Mexico; and English, Ateso, Jopadhola and Runyoro in Uganda) using interview guides described in Appendix 1, 2 and 3. Debriefing sessions with the entire research team will be held each week. Interviews will take place over phone/zoom/skype or in-person depending on the local situation of COVID-19 pandemic and will be audio recorded. Participants will be contacted at the health units or their office (policy makers) and will be interviewed in an area within the unit that meets the appropriate privacy conditions. We aim to conduct up to 60 interviews in each country (180 interviews in total).

Data analysis: Interviews will be transcribed verbatim in-country and transcripts in Hindi, Telugu, Spanish, and Ateso, Jopadhola and Runyoro will be translated to English for analysis. The qualitative data for each country will be coded using NVivo software (QRS International, Vic) and analysed using an inductive approach. Two coders from each country will review and analyse the data. Weekly calls will be set up to discuss the emerging themes with the research team. This approach will enable us to explore and identify the important issues in PHC workforce organisation, composition and comprehensiveness, and will also help us to identify shared challenges and differences across countries.

For peer review only - http://bmjopen.bmj.com/site/about/guidelines.xhtml

Triangulation of data: The emergent themes from the qualitative interviews in each country will be interpreted in conjunction with the SARA survey and outputs from the policy analysis. Data integration of the three objectives will help us identify the policy and implementation gaps for each country.

Objective 4: Conduct a comparative analysis of the relationship between PHC team composition and organisation with the delivery of comprehensive PHC across the three countries.

Data analysis: We will use a case-oriented research strategy where each 'case' (country) will be considered analytically as a whole. [24,25] Comprehensiveness of services (which services such as prevention, promotion, treatment, rehabilitation or palliation; for what conditions and by whom) will be explored through the policy review, SARA (availability of infrastructure to deliver CPHC) and interviews with PHC team members. Cross country comparisons will be conducted to understand similarities and differences in PHC related policies, especially in terms of the workforce composition, organisation and service delivery with the intention of *learning about* the different approaches to CPHC and PHC workforce organisation, the *context* in which PHC systems exist, and *why* they take the forms they do. The comparison will examine the differences and similarities between PHC policies, organisation, and service delivery in the three countries.[25]

ETHICS

Ethical approvals have been sought from Institutional Ethics Committee of The George Institute for Global Health, India for the Indian sites (Ref 16/2020); School of Medicine Research Ethics Committee at Makerere University for the sites in Uganda (Ref 2020-218); and the Mexican National Institute of Public Health (INSP for its Spanish acronym) ethics review board for the sites in Mexico (Ref: 1726). Additional permissions have been sought from the Uganda National Council for Science and Technology and Tororo and Buliisa District Health Offices and the INSP Research committee. The local health authorities will provide approval for collection of data at the facility level. The respondents will be adequately informed regarding all relevant aspects of the study, including its aim and interview procedures, through a participant information sheet. Respondents who accept to participate in the study will provide signed written informed consent. All participants will be given written participant

For peer review only

For peer review only - http://bmjopen.bmj.com/site/about/guidelines.xhtml

information sheets prior to consenting to participate in this study. Data collection instruments will be piloted and administered by means of electronic questionnaires on mobile devices. We anticipate that data collection for SARA and semi-structured interviews will take approximately 12 months and analysis for the entire study will take additional six months. As data collection is taking place during the COVID-19 pandemic, provision to conduct interviews online has been made accordingly.

SIGNIFICANCE

This study will provide insight into the availability of policies on PHC, the implementation of policies on PHC workforce, team organisation and service provision for the delivery of CPHC. Furthermore, it will investigate how different LMICs organise their PHC teams to deliver UHC through comprehensive primary care.

For peer review only

Author's contribution

The study was designed by RJ, DP, and DeP. The first draft was written by RJ, IB, DeP with inputs from RM, FGS and DP. MS, IBH-P, HR-M, ReJ, ES-M, LA-B, EO-N, DO-A, NJ, OJ, RaJ, SU, VA, DeP provided inputs to the protocol. All authors have read and approved the final manuscript.

Funding

This study is funded by the Bill and Melinda Gates Foundation through the Primary Health Care Research Consortium hosted at the George Institute for Global Health in India. RJ is funded by the National Heart Foundation of Australia (Grant number 102059) and the University of New South Wales Scientia Fellowship (No grant number).

Acknowledgement

We would like to acknowledge the Primary Health Care Research Consortium and the team in Uganda (Midiam Ibáñez-Cuevas, Carlos Chivardi-Moreno, Namatovu Jane, and Onyango Jude) for their contribution. This work was supported, in whole, by the Bill & Melinda Gates Foundation [INV-000970]. Under the grant conditions of the Foundation, a Creative Commons Attribution 4.0 Generic License has already been assigned to the Author Accepted Manuscript version that might arise from this submission.

Declaration of Interests

The researchers declare that they have no conflicts of interest.

For peer review only

REFERENCES

1 Bitton A, Ratcliffe HL, Veillard JH, et al. Primary Health Care as a Foundation for Strengthening Health Systems in Low- and Middle-Income Countries. *J Gen Intern Med* 2017;32(5):566-71.doi:10.1007/s11606-016-3898-5 [published Online First: 13 December 2016]

2 Bitton A, Fifield J, Ratcliffe H, et al. Primary healthcare system performance in low-income and middle-income countries: a scoping review of the evidence from 2010 to 2017. *BMJ Glob Health* 2019;4(Suppl 8):e001551.doi:10.1136/bmjgh-2019-001551

3 Goodyear-Smith F, Bazemore A, Coffman M, et al. Primary care financing: a systematic assessment of research priorities in low- and middle-income countries. *BMJ Glob Health* 2019;4(Suppl 8):e001483.doi:10.1136/bmjgh-2019-001483

4 Stenberg K, Hanssen O, Bertram M, et al. Guide posts for investment in primary health care and projected resource needs in 67 low-income and middle-income countries: a modelling study. *Lancet Glob Health* 2019;7(11):e1500-e10.doi:10.1016/S2214-109X(19)30416-4

5 World Health Organization, United Nations Children's Fund. A vision for primary health care in the 21st century: towards universal health coverage and the Sustainable Development Goals. Geneva: World Health Organization, 2018.

6 World Health Organization. Building the economic case for primary health care: a scoping review. Geneva: World Health Organization, 2018.

7 Angell B, Dodd R, Palagyi A, et al. Primary health care financing interventions: a systematic review and stakeholder-driven research agenda for the Asia-Pacific region. *BMJ Glob Health* 2019;4(Suppl 8):e001481.doi:10.1136/bmjgh-2019-001481

8 Veillard J, Cowling K, Bitton A, et al. Better Measurement for Performance Improvement in Low- and Middle-Income Countries: The Primary Health Care Performance Initiative (PHCPI) Experience of Conceptual Framework Development and Indicator Selection. *Milbank Q* 2017;95(4):836-83.doi:10.1111/1468-0009.12301 [published Online First: 2017/12/12]

For peer review only - http://bmjopen.bmj.com/site/about/guidelines.xhtml

9 Muldoon LK, Hogg WE, Levitt M. Primary care (PC) and primary health care (PHC). What is the difference? *Can J Public Health* 2006;97(5):409-11.doi:10.1007/bf03405354 [published Online First: 2006/11/24]

10 World Health Organization. Building the primary health care workforce of the 21st century. Geneva: World Health Organization, 2018.

11 World Bank Group, World Health Organization, Ministry of Finance P.R.C., et al. Deepening Health Reform in China: Building High-Quality and Value-Based Service Delivery. Washington, DC: World Bank, 2016.

12 Rodriguez HP, Rogers WH, Marshall RE, et al. Multidisciplinary primary care teams: effects on the quality of clinician-patient interactions and organizational features of care. *Med Care* 2007;45(1):19-27.doi:10.1097/01.mlr.0000241041.53804.29 [published Online First: 2007/02/07]

13 Afsana K, Alam MA, Chen N, et al. Exemplars in Global Health - Community Health Workers in Bangladesh 2021 [Available from: https://www.exemplars.health/topics/community-health-workers/bangladesh, accessed 8 December 2020].

14 Vasan A, Mabey DC, Chaudhri S, et al. Support and performance improvement for primary health care workers in low- and middle-income countries: a scoping review of intervention design and methods. *Health Policy Plan* 2017;32(3):437-52.doi:10.1093/heapol/czw144 [published Online First: 2016/12/21]

15 Wranik WD, Price S, Haydt SM, et al. Implications of interprofessional primary care team characteristics for health services and patient health outcomes: A systematic review with narrative synthesis. *Health Policy* 2019;123(6):550-63. doi: 10.1016/j.healthpol.2019.03.015 [published Online First: 2019/04/09]

16 Starfield B, Shi L, Macinko J. Contribution of primary care to health systems and health. *Milbank Q* 2005;83(3):457-502. doi: 10.1111/j.1468-0009.2005.00409.x [published Online First: 2005/10/06]

17 Primary Health Care Research Consortium. Primary Health Care Research Consortium (PHCRC) 2020 [Available from: https://phcrc.world/, accessed December 9 2020].

For peer review only - http://bmjopen.bmj.com/site/about/guidelines.xhtml

18 Ministry of Health & Family Welfare India. Ayushman Bharat: Comprehensive Primary Health Care through Health and Wellness Centers - Operational Guidelines. New Delhi: Government of India, 2018.

19 González Block MÁ, Reyes Morales H, Cahuana Hurtado L, et al. Mexico: Health system review. *Health Syst Transit* 2020;22(222)

20 Ssengooba F, Rahman SA, Hongoro C, et al. Health sector reforms and human resources for health in Uganda and Bangladesh: mechanisms of effect. *Hum Resour Health* 2007;5(1):3. doi: 10.1186/1478-4491-5-3

21 Murindwa G, Tashobya CK, Kyabaggu JH, et al. Meeting the challenges of decentralised health service delivery in Uganda as a component of broader health sector reforms. In: Christine Kirunga Tashobya, Freddie Ssengooba, Valeria Oliveira Cruz, ed. Health Systems Reforms in Uganda: Processes and Outputs. UK: Health Systems Development Programme, London School of Hygiene & Tropical Medicine 2006:97-109.

22 Primary Health Care Performance Initiative. The PHCPI Conceptual Framework 2018 [Available from: https://improvingphc.org/phcpi-conceptual-framework, accessed 1 June 2021].

23 Primary Health Care Performance Initiative. Team-Based Care Organization 2018 [Available from: https://improvingphc.org/improvement-strategies/facility-organization-and-management/team-based-care-organization, accessed 1 June 2021].

24 Imbrogno S. A case oriented research strategy as a comparative method. *J Int Comp Soc Policy* 1994;10(2):137-48.doi:10.1080/17486839408414909

25 Cacace M, Ettelt S, Mays N, et al. Assessing quality in cross-country comparisons of health systems and policies: towards a set of generic quality criteria. *Health Policy* 2013;112(1-2):156-62.doi:10.1016/j.healthpol.2013.03.020 [published Online First: 2013/05/01]

26 World Bank. GDP per capita, PPP (current international $) 2021 [Available from: https://data.worldbank.org/indicator/NY.GDP.PCAP.PP.CD., accessed 1 June 2021]

For peer review only - http://bmjopen.bmj.com/site/about/guidelines.xhtml

27 World Health Organization. Life expectancy and healthy life expectancy Data by country 2020 [Available from: https://apps.who.int/gho/data/view.main.SDG2016LEXv?lang=en., accessed 1 June 2021]

28 World Health Organization. Maternal mortality Estimates by country 2019 [Available from: https://apps.who.int/gho/data/view.main.1390., accessed 1 June 2021]

29 Mor N, Kalita A. Financing Universal Health Care in India. *SSRN Electronic Journal* 2015 doi:10.2139/ssrn.2606918

30 Lahariya C. Health & Wellness Centers to Strengthen Primary Health Care in India: Concept, Progress and Ways Forward. *Indian J Pediatr* 2020;87(11):916-29.doi:10.1007/s12098-020-03359-z [published Online First: 2020/07/09]

31 Shamah-Levy T, Vielma-Orozco E, Heredia-Hernandez O, et al. Encuesta Nacional de Salud y Nutricion 2018-19: Resultados Nacionales. Cuernavaca, Mexico: Instituto Nacional de Salud Publica, 2020.

For peer review only

For peer review only - http://bmjopen.bmj.com/site/about/guidelines.xhtml

Table 1. Primary Health Care context in India, Mexico and Uganda.

	India	Mexico	Uganda
Population, 2020	1.38 billion	128 million	45 million
GDP per capita, PPP (current international $), 2019 [26]	6996.56	20,944.03	2,284.27
Life expectancy at birth (years), 2019 [27]	70.8	76.0	66.7
Maternal mortality ratio (per 100 000 live births), 2017 [28]	145	33	375
Under-five mortality rate (deaths per 1000 live births), 2020 [28]	35.7	16	57.1
Organization	Three tired system:<br>• Sub-center the most peripheral and first contact point between the community and health system<br>• PHC is the first contact point between village community and the Medical Officer<br>• Community Health Center with specialized medical and paramedical staff is the referral unit for PHCs<br>• Tertiary level includes hospital and medical colleges	Three level public health system in Health Districts:<br>• Community Health Centers with a medical doctor student in social service and a nurse<br>• Integrated Community Health Centers with health personnel, nurses, medical doctors, nutritionist, physical activator, social worker<br>• Secondary level includes general hospitals and staff of medical specialties is the referral unit for CHCs<br>• Tertiary level includes hospital of high specialties	Five-level system with health centres I, II, III, IV and the general hospital being the apex of the PHC system. All this functions with the Health Sub District administrative system
Financing	• In 2015-16, 43% of out of pocket expenditure by households was done on primary care.[29]<br>• National Health Policy 2017 commits a major proportion (>2/3rds) of resources to PHC	• In 2018, 50% of total health spending came from Government schemes and compulsory contributory health care financing schemes, of which 24% was spent on primary care units.<br>• 42% of total health spending was out-of-pocket<br>• Population with household expenditures on health greater than 10% of total household expenditure or income (SDG indicator 3.8.2) 1.5%	Uganda's out of pocket on primary care increased through 38.4% through a period of 2004-2018.<br>The government of Uganda expenditure on health has stagnated at around 9.6% of its GDP with regards to the Abuja declaration of 15%

For peer review only - http://bmjopen.bmj.com/site/about/guidelines.xhtml

CPHC	The Health and Wellness Center (HWC) component of Ayushman Bharat Programme aims to provide comprehensive primary health care by upgrading and making 150,000 existing sub-centers and primary health centers functional by December 2022.[18] The first HWC was launched on 14 April 2018 and by 31 March 2020, a total 38,595 AB-HWCs were operational across India.[30]	By 2018, 19% of population have no Universal Health Coverage,[31] and to solve this, The National Health Plan 2018-2024 create the Health Institute for Welfare, component of Mexican Health System, and aims to provide comprehensive primary health care by organizing health districts based in geographical areas by 2024.	The Uganda National Minimum Health Care Package (UNMHCP) comprises of interventions that address major causes of morbidity and mortality both communicable and non-Communicable diseases including disease prevention and health promotion. This package of services is funded by government

For peer review only

For peer review only - http://bmjopen.bmj.com/site/about/guidelines.xhtml

Figure 1. Primary Healthcare Performance Initiative (PHCPI) conceptual framework

Figure 2. Mixed-methods study design

Figure 3. Sample level distribution and methodological approach

For peer review only

For peer review only

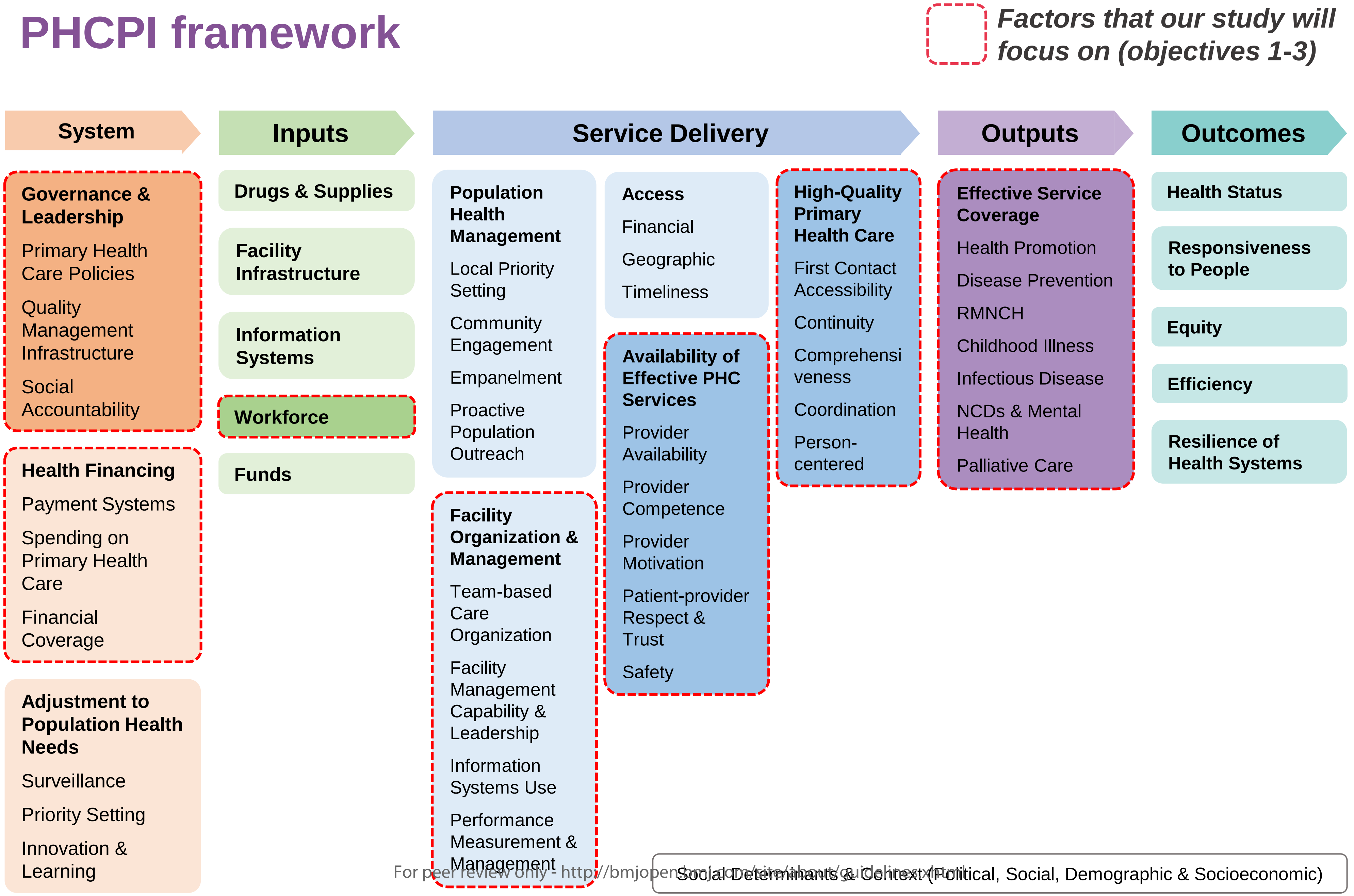
PHCPI framework
Factors that our study will focus on (objectives 1-3)
System
Inputs
Service Delivery
Outputs
Outcomes
Governance & Leadership
Primary Health Care Policies
Quality Management Infrastructure
Social Accountability
Health Financing
Payment Systems
Spending on Primary Health Care
Financial Coverage
Adjustment to Population Health Needs
Surveillance
Priority Setting
Innovation & Learning
Drugs & Supplies
Facility Infrastructure
Information Systems
Workforce
Funds
Population Health Management
Local Priority Setting
Community Engagement
Empanelment
Proactive Population Outreach
Facility Organization & Management
Team-based Care Organization
Facility Management Capability & Leadership
Information Systems Use
Performance Measurement & Management
Access
Financial
Geographic
Timeliness
Availability of Effective PHC Services
Provider Availability
Provider Competence
Provider Motivation
Patient-provider Respect & Trust
Safety
High-Quality Primary Health Care
First Contact Accessibility
Continuity
Comprehensiveness
Coordination
Person-centered
Effective Service Coverage
Health Promotion
Disease Prevention
RMNCH
Childhood Illness
Infectious Disease
NCDs & Mental Health
Palliative Care
Health Status
Responsiveness to People
Equity
Efficiency
Resilience of Health Systems
Social Determinants & Context (Political, Social, Demographic & Socioeconomic)

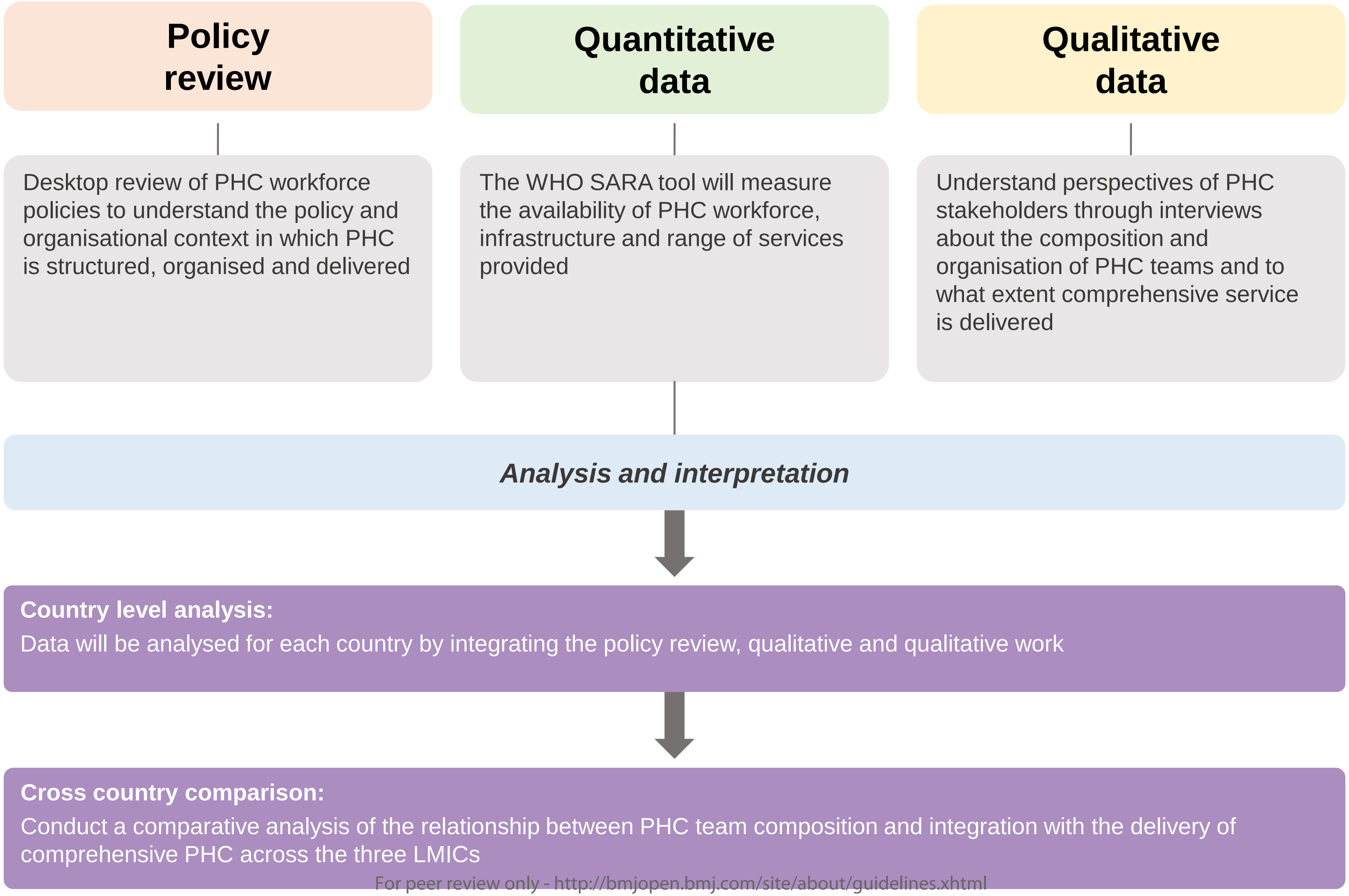

For peer review only - http://bmjopen.bmj.com/site/about/guidelines.xhtml

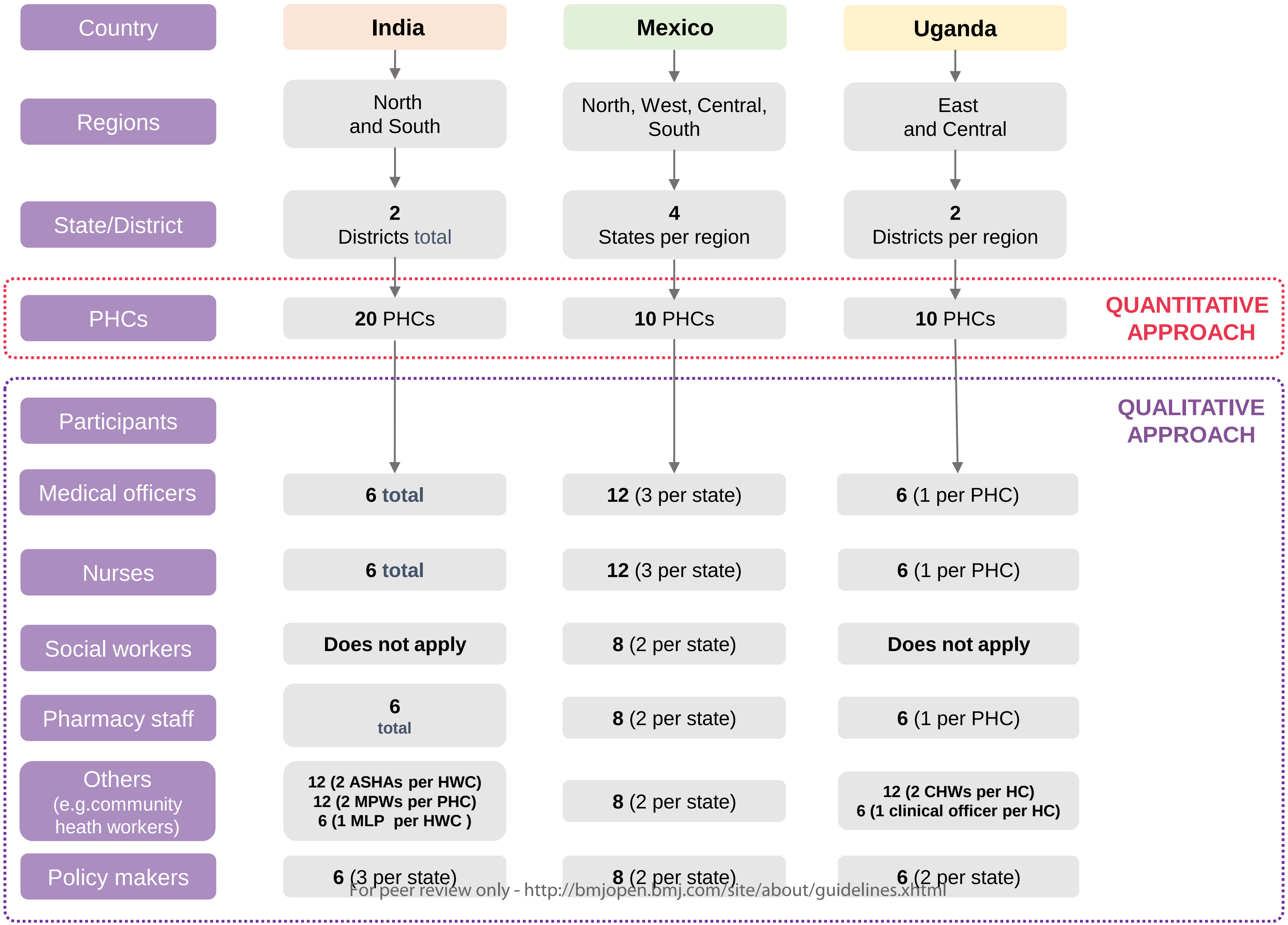

For peer review only - http://bmjopen.bmj.com/site/about/guidelines.xhtml

In-depth interview guide with policy makers (National & regional heads, program coordinators)

Main question/exploratory questions	Probing questions
**Role and recruitment**<br>How are the following cadres of Primary Health Care workers recruited?<br>- Doctors<br>- Nurses/midwives<br>- Community Health Workers (Community Health Officers, Community Health Extension Workers and Junior Community Health Extension Workers)<br><br>What is expected from Primary Health Care workers to delivery comprehensive primary health care (including NCDs)<br>How do you support Primary Health Care workers to deliver high quality service?	Who selects them? How?<br>How are they posted?
**Training**<br>What is your view about Community Health Worker's training in the implementation of comprehensive primary health care?<br><br>What are the challenges faced in training the Primary Health Care workers to provide comprehensive primary health care?<br><br>What is the plan for their continuous training to equip the Primary Health Care workers to deliver comprehensive primary health care	Do you think the training is adequate to deliver comprehensive primary health care? (communicable and non-communicable diseases prevention and management?<br>Community Health Workers: How do you evaluate the quality of the training given by the school of health technologies health sciences colleges? (modify this based on the country's CHW program)<br>Do you think the Community Health Workers have a capacity to provide care for conditions other than MCH?<br>What plans are there to teach new skills on a regular basis (e.g. fortnightly or quarterly mentorship or on-the-job training)?

For peer review only

**Accreditation**<br>What is the system of accreditation for the level Community Health Workers?	How is the accreditation system implemented? What happens if minimum standards are not met prior to practicing? How could accreditation be improved?
**Equipment and Supplies**<br>Do you think there is optimal provision of equipment and supplies for the Primary Health Care workers to provide comprehensive primary health care?<br><br>What challenges do you think health facilities faced in delivering services?	What protocols, guidelines, and other teaching aid are available for the non-communicable diseases program at the health facility level?
**Supervision**<br>How are the Primary Health Care workers supervised	What is the role of the Federal/State/LGAs in the supervision of the Primary Health Care workers?<br>Is there any particular strategy implemented for the supervision process?<br>Which activities are focussed on during<br>Is there a system to provide supervision feedback/ reports to the Primary Health Cares?
**Team work**	
How is work distributed among the team?	Are tasks shared between different cadres of the workforce?
**Incentives**<br>How are Primary Health Care workers remunerated for their work?<br><br><br><br>**Retention and motivation**<br>What are the common reasons why Primary Health Care workers leave their work?<br>What strategies are in place to reduce the attrition of the Primary Health Care workers and ensure adequate distribution, and improve their motivation?	Are salaries processed on time?<br><br>Are there any challenges with payment processes?<br><br>Are there any additional incentive for implementing non-communicable diseases related or other programme activities<br><br>Retention and motivation<br>- Reasons: administrative reasons (work-related reasons), financial reasons, social reasons (family issue, pursue higher education, not recognized or valued by the community)
**Community Involvement** What strategies are in place for the community to support the Community Health Worker in the implementation of NCD programmes?	- What work has been done/planned to introduce the non-communicable diseases implementation role of

	Community Health Workers to the community?
**Opportunity for advancement**<br>What are the opportunities for further promotion or professional advancement through the Primary Health Care workers?	What are the challenges on to achieving this?<br><br>Are there any method to evaluate individual performance?<br><br>Is performance linked with opportunities for career progression?
**Data**<br>Are any strategies being used to improve information management systems through at the Primary Health Care?	How do supervisors monitor the quality of documents and provide assistance?<br>How are information transmitted from the health facility to other part of the health system
**Linkages to Health System**<br>Referral System:<br>How does the referral system work?	Do you think is there a clear referral guideline for the Primary Health Care workers to refer patients?<br>- Are there feedback mechanisms in place<br>- Any arrangement for the emergency conditions
**General questions**<br>What are your biggest challenges for delivering Comprehensive Primary Health Care?	What changes would you recommend?

For peer review only

In-depth interview guide for Community Health Workers

Main question/exploratory questions	Probing questions
**Role and recruitment**<br>Could you let know about the process of your recruitment?<br><br>**Role of the Community Health Workers**<br>What is your role/job description?<br><br>What is expected from you in performing this role?	 <br><br><br>What service do you provide?<br><br>Any challenges: work load, lack of skill?
**Training**<br>Could you tell me about the training received?<br>Did you get evaluated at the end of your training?<br>Do you receive ongoing training/refreshment training to keep your skills up to date?	Do you feel you had adequate training for MCH, infectious and chronic diseases prevention and management?<br><br>How confident do you feel to provide chronic diseases services?
**Accreditation**<br>Is your training/ course accredited?	Is there any system of accreditation?
**Equipment and Supplies**<br>Do you have supplies and equipment you need for providing services?	What about the equipment for the chronic diseases care?<br>Do you receive protocols and guidelines for implementation of the programmes?<br>Do you use any digital technologies like mobile apps or SMS to assist your work?
**Supervision**<br>Can you describe how you are being supervised?<br><br>Do you feel supported? E.g. if there is any error committed in the field, is there someone you can turn easily?	Who supervises you?<br>How often does your supervisor visit you? Is it enough?<br>What does your supervisor do when he/she visits you?<br>○ Observation of service delivery

For peer review only

For peer review only - http://bmjopen.bmj.com/site/about/guidelines.xhtml

	○ Coaching and skills development<br>○ Trouble shooting, problem solving<br>○ Record Review<br>○ Supply check<br>Do you receive any feedback regarding your supervision?<br>Do you think this type of supervision is helpful?
**Team**<br>Do you feel like you are part of a larger team?	How often do you interact with colleagues in the Primary health centre?
**Incentives**<br>What do you think about your remuneration?<br><br>What are the common reasons that Community Health Workers leave their work?<br>What incentives do you think would motivate Community Health Workers and retain them in their job?	Salaries are consistently paid on time.<br>Increase in salary based on performance?<br><br>Ask about administrative reasons (work-related reasons), financial reasons, social reasons (family issue, pursue higher education, being not recognized or valued by the community)
**Community Involvement** Does the community provide any support to you?<br>Do you engage the community in chronic diseases services provision?	Do they provide feedback, support (like financial/gifts in kind), formal recognition/appreciation?
**Opportunity for advancement**<br>What are the opportunities for further promotion or professional advancement in your career?	In what way do you prefer to pursue your career development?
**Data**<br>Tell me about how the information management system works in your facility	What do you do with the data you collect on chronic diseases in your facility?<br>What process is in place to ensure quality of data and provide assistance when needed?
**Linkages to Health System**<br>**Referral System**<br>How do you refer patients for health services you do not or cannot provide?	Do you need to complete a referral form for the client to take to the facility?

For peer review only - http://bmjopen.bmj.com/site/about/guidelines.xhtml

- What about for chronic diseases?	Do you receive any feedback or counter referral from the facility for patients you have referred?<br><br>How do you refer patient with cardiovascular diseases/ Diabetes/ suspected chronic disease cases?
**General questions**<br>What are your biggest challenges as a Community Health Worker?	What changes are needed to help you do your job better?

For peer review only

For peer review only - http://bmjopen.bmj.com/site/about/guidelines.xhtml

In-depth interview guide with health care workers working in the primary healthcare facilities

Main question/exploratory questions	Probing questions
**Role and recruitment**<br>In your opinion, what kind of strategies are needed to ensure delivery of comprehensive primary health care?	What is your view about the role of Community Health Workers in chronic disease control?
**Training**<br>What is your view about Community Health Workers' training?	Do you think Community Health Workers have sufficient knowledge and skill to implement comprehensive primary health care?
**Equipment and Supplies**<br>Do you think there is enough equipment and supplies to adequately manage acute and chronic diseases at Primary Health Centre level	Are protocols, guidelines for the chronic disease programs adequately implemented?<br><br>What are the challenges to improving adherence to guidelines and protocols?<br><br>What kind of equipment and supplies should be provided?
**Supervision**<br>What is your view of the Community Health Workers supervision process?	What is your/your facilities role in the Community Health Workers' supervision?<br><br>What kind of supervision should be in place for Community Health Workers to strengthen the delivery of comprehensive primary health care?
**Incentives**<br>What factors do you think influence Community Health Workers to remain in or leave their job?	Administrative reasons, financial reasons, social reasons
**Community Involvement** How involved is the community in the management of the Health Facility?	Why/ why not?

For peer review only - http://bmjopen.bmj.com/site/about/guidelines.xhtml

**Data**<br>Are the information management systems in your facility adequate?	What is needed to overcome challenges to better information management?<br>Who is responsible for data management?<br>How are the data from your facility managed?
**Linkages to Health System Referral System**<br>Can you describe the referral processes?<br><br>What is the challenge for having a good linkage between the health post and the health services?	Are referral guidelines for Community Health Worker?<br><br>What would you recommend for improving linkage of services from the community to the primary healthcare system?
**Integration of chronic diseases program**<br>How integrated are chronic disease programmes with other activities in your Primary Health Centre?	What is your recommendation for effective integration of chronic disease services in the Primary Health Centre settings?<br>What will be the challenges?

For peer review only
